# Qxpak.5: Old mixed model solutions for new genomics problems

**DOI:** 10.1186/1471-2105-12-202

**Published:** 2011-05-25

**Authors:** Miguel Pérez-Enciso, Ignacy Misztal

**Affiliations:** 1 ICREA - CRAG - Universitat Autònoma de Barcelona, 08193 Bellaterra, Spain; 2 Department of Animal and Dairy Science, University of Georgia, Athens, GA 30602, USA

## Abstract

**Background:**

Mixed models have a long and fruitful history in statistics. They are pertinent to genomics problems because they are highly versatile, accommodating a wide variety of situations within the same theoretical and algorithmic framework.

**Results:**

Qxpak is a package for versatile statistical genomics, specifically designed for sophisticated quantitative trait loci and association analyses. Multiple loci, multiple trait, infinitesimal genetic effects, imprinting, epistasis or sex linked loci can be fitted. The new version (v. 5) allows us, among other new features, to include either relationship matrices obtained with molecular information or user defined matrices that can be read from an input file. This feature can be used for genome selection or - more importantly - to correct for population structure in association studies. In crosses, two parental lines, not necessarily inbred, can be accommodated.

**Conclusions:**

This software aims at simplifying statistical genetic analyses implementing a coherent and unified approach by mixed models. It provides a tool that can be used in a wide variety of situations with ample genetic and statistical modeling flexibility. The software, a complete manual and examples are available at http://www.icrea.cat/Web/OtherSectionViewer.aspx?key=485&titol=Software:Qxpak.

## Background

Mixed models (MM) have a long and fruitful history in statistics, and in statistical genetics in particular. They are called mixed because both 'fixed' and 'random' effects are included in the model. Their earliest and most successful application in genetics, was in BLUP (best linear unbiased prediction) of genetic merit. Most of genetic progress in all animal species since the early 1980's is due to MM [1]. In this application, the genetic merit of animals is assumed to be a random variable, normally distributed with variance proportional to the heritability (h^2^) of the trait, whereas the environmental - confounding - effects are typically fitted as fixed effects.

Much more recently, MM have received renewed attention by the human genetics community because they are a powerful tool to correct for population structure [2,3]. These authors actually show that MM perform better than competing principal component analysis approach. Structure is one of the most frequent causes for false positives in genome wide association studied (GWAS), so the relevance of mixed models to genomics cannot be underestimated. Currently, there is intense discussion on the 'hidden heritability', i.e., the discrepancy between estimated h^2 ^and the proportion of variance explained by individual loci, the latter being much smaller usually than the former. Again using MM technology, Yang et al. [4] have shown that this discrepancy can be explained by lack of power and SNP ascertainment bias.

Mixed models are pertinent to genomics problems because they are highly versatile. They can accommodate a wide variety of situations within the same theoretical and algorithmic framework. In the applications to correct for population structure mentioned above [2,3], a multivariate normal random effect with covariance the molecular relationship matrix is incorporated in the model, whereas the net effect of the SNP is treated as fixed. Therefore, classical algorithms can be used and the same theoretical properties of the estimators are expected. Further, we anticipate that polymorphism based matrices to quantify genetic relationship will be a key instrument to analyze association studies when complete sequence is available, and MM will continue to be used in this setting.

Over the last years, we have been implementing mixed model approaches to deal with genomics problems, and these have been incorporated in the package Qxpak [5]. Although an array of softwares for QTL analyses are already implemented, e.g., R/qtl [6], Plink [7], GenABel [8] or EMMAX [3] or FunMap [9] for functional analyses, Qxpak is complementary to these or other packages. Specifically, Qxpak offers a unique flexibility in statistical modeling. For example, Qxpak accommodates multi-trait models where models can differ from trait to trait. Each trait can be modeled combining different relationship matrices, including pedigree and marker based matrices or, importantly, allowing for any pattern of missing data.

The original software was aimed primarily at dealing with Quantitative Trait Locus (QTL), that is, linkage analyses. Here we present a new version (Qxpak.5) that incorporates important advances with respect to the first version to adapt to current large-scale genomic datasets. In addition to improved algorithms, the main addition are: full implementation of association analyses where the marker (e.g., SNP) can be treated either as fixed or random. Also, epistasis and imprinting gene actions are implemented, as is computing the marker coancestry matrix to allow for linkage disequilibrium (LD) analyses in structured populations. Besides, Qxpak.5 allows us to include any user-defined covariance and/or dominance relationship matrix. A complete list of new options is in the manual available at the website.

## Implementation

Qxpak relies on the well known theory of mixed models as applied to a variety of genomics problems. A general mixed model is of the type(1)

where **y **is a vector containing the recorded performances, **b **contains the fixed effects to be estimated, **g_k _**contains the genetic (QTL) effects for any of the *N_q _*QTL affecting the trait. Finally, **X **and **Z **are incidence matrices that relate observations to the parameters in the **b **and **g **vectors, and **e **is a vector of residuals.

Mixed model theory dictates that the distribution of random variables, i.e., their means and variances, must be specified. It is by establishing the distribution of the genetic effects that MM offers complete flexibility to adapt to a variety of genomics issues.

**Figure 1:**
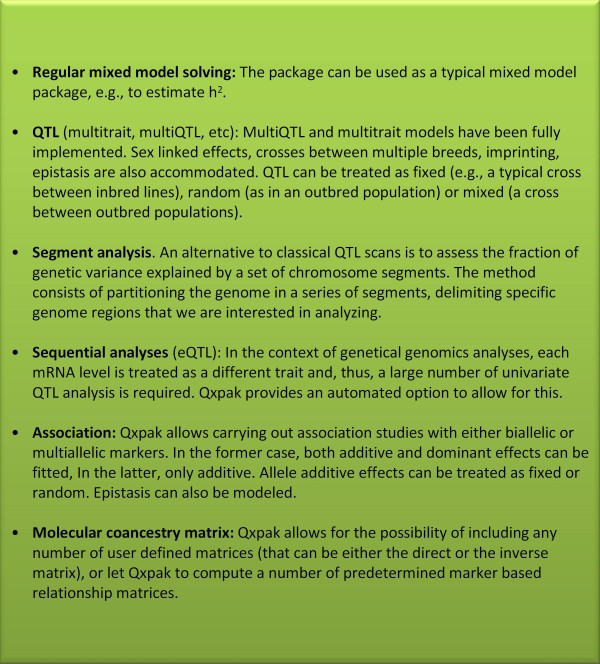
**The main kind of analyses that Qxpak.5 can be used for**.

The main kind of analyses that Qxpak can perform are (see also Figure 1, whereas theoretical details are in the additional file 1):

• *Linkage analyses in crosses *(QTL): The expectation of *g *depends on the probability that the locus is identical by descent to each of the founder breeds. Qxpak can analyze crosses between several breeds and any design, e.g., backcross and F_2_, F_3_,... data can be analyzed jointly. If the lines are assumed inbreed, **g **is treated as fixed. Otherwise, the covariance matrix is a weighted average of probabilities that two individuals are identical by descent (IBD) for alleles originating in each of the founder breeds [10].

• *Polygenic effect*: In this case, **g **~ N(**0**, **A**), where **A **is the numerator relationship matrix; **A **quantifies the probability that two individuals are IBD conditional on pedigree information, i.e., two individuals whose parents are unknown are taken to be unrelated [1].

• *Linkage analyses in outbred populations: *Here **g **~ N(**0**, **G**), where **G **is a covariance matrix which elements are the probabilities that individuals are IBD *conditional *on pedigree and marker information.

• *Linkage Disequilibrium (LD): *Now *g *~ N(λa, ), where λ is an indicator variable that depends on the genotype at the SNP, 1 and -1 for homozygous, 0 for heterozygous, and  is the variance associated with the locus. In general, though, *g *is taken as fixed in LD analyses. Qxpak allows for both options, either fixed or random.

• *Molecular coancestry*: It has been known for quite some time that genome wide association analyses are prone to false positives, particularly in structured populations [2,11-13]. Qxpak implements the use of molecular coancestry matrix, as suggested by several authors, e.g., [4,14]. Two options are available, either raw or standardized covariance matrices. In the first case, **G **= **MM'**/n, where **M **is a *m *individuals × *n *SNPs matrix with values -1, 0 and 1 for genotypes 11, 12 and 22, respectively. Note that **MM' **contains the scaled number of alleles shared between individuals, averaged over markers. In the standardized matrix, G* = **WD^-1^W'**/n, where **W **= {*w_ij _*= (*m*_ij _- *μ_j_*)} where *μ_j _*is the genotypic mean frequency of i-th SNP (2*q*_i _-1), *q*_i _being the frequency of allele 2, and **D **is a diagonal matrix with elements 2 *q*_i _(1- *q*_i_), the genotype variance. This is the option considered, e.g., in Yang et al. [4] except that they employ a slightly different variant for the diagonal elements.

Qxpak allows to combine any number of loci, where each one can be modeled differently. For instance, a genome wide LD scan can be carried out by including also a polygenic effect. Alternatively, a combined linkage/LD analyses can be implemented [15].

Complex genetic effects like imprinting, epistasis or interaction between sex and QTL can be accounted for in Qxpak. Imprinting means that only either the paternal or the maternal allele is expressed. Although only a small percentage of genes are known to undergo imprinting, this phenomenon can be of importance in some complex traits, e.g., IGF2 gene effect on growth in pigs [16]. Therefore, it can be worthwhile to allow for imprinting in QTL or association analyses. Qxpak does that by modifying how IBD probabilities are computed. In an LD analysis, Qxpak identifies whether the paternal or maternal origins can be determined using parents' genotypes. If the parental status of the alleles cannot be determined, this genotype does not contribute to total likelihood. The indicator variable *λ *is fitted according to the paternal allele for maternal imprinting (and vice versa). Note that, with imprinting, dominance is not defined. In the case of epistasis, Qxpak permits some predetermined options or user defined interactions. It also allows for epistasis between random QTL effects.

**Figure 2:**
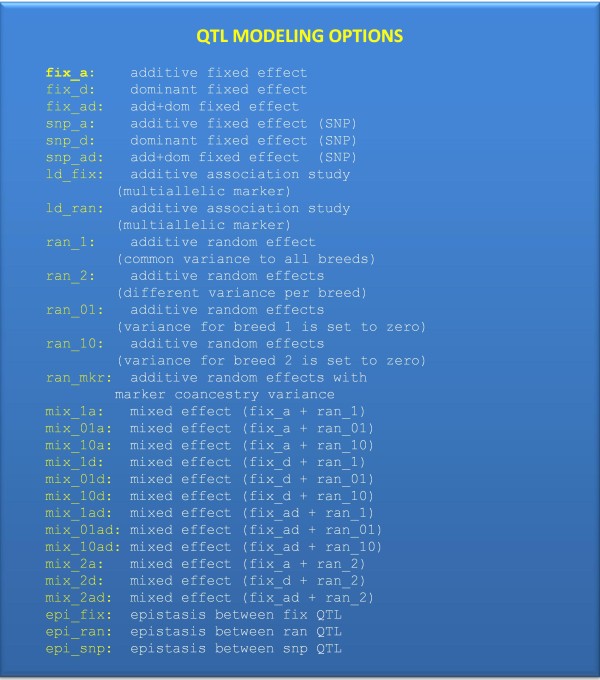
**All available QTL modeling options**. Each QTL can be modeled differently. In addition, random effects can be modeled as polygenic effects or following a user defined covariance matrix.

Qxpak is implemented in fortran95 to take advantage of specialized sparse matrix software [5]. Typically, input requires a parameter file, a data file containing phenotypes and any effect that may be included in the model, a pedigree file and a marker file with genotypes. The parameter file contains different sections (see complete manual). The main ones are the QTL section, where genetic effects are defined (Figure 2) and the TRAIT section, where the models to analyze each phenotype are specified. Not all effects present in the data file need to be included in the model, i.e., the same files can be used to run a number of different analyses. The output provides solutions of both fixed and random effects, residuals if requested, and P-values of the test (e.g., the QTL). Figure 2 describes the different approaches to model a QTL in Qxpak.

The program computes, if required, the IBD relationship between individuals given marker and pedigree information. These coefficients are computed using MCMC methods; this is the most computationally expensive step, but files are saved and reused automatically for successive analyses. REML/ML estimates of variance components are obtained via the EM (expectation maximization). Timing for an example data set is shown in Table 1.

**Table 1:** Performance in server with processor Xeon D5060 3.2 GHz (CPU time)

Step	Linkage dataset (N = 780, 187 microsats)	GWAS dataset (N = 560, 39,209 SNPs)
Computing IBD coefficients	15'	-
QTL scan	1'	30'
Scan with infinitesimal genetic effect	15'	3 h19'

## Results: Application to F2 cross

Here we show by proof of example how Qxpak can be used to gain insight into the genetic architecture of a complex trait. We reanalyzed ~ 500 carcass length measurements from an Iberian × Landrace intercross that comprises F2, F3 and backcross individuals [17]. The original markers were 11 microsatellites in chromosome 4 that were binarized here such that allele frequencies were quite dissimilar in each parental breed, markers were equally spaced every 5 cM. We considered different models that are listed in Table 2. In the first comparison we omitted or fit an infinitesimal effect (Models 1 and 2). In Qxpak, an infinitesimal effect is fitted in the EFFECT section of the parameter file as

**Table 2:** Effect estimates with different models for carcass length data from an Iberian × Landrace intercross. Fixed effects include sex, batch and ham weight

Model	*u *()	q_a_	q_d_	q_a_(sex)	snp
0	0.38	-	-	-	-
1	-	-0.96 ± 0.19	-	-	-
2	0.39	-0.97 ± 0.19	-	-	-
3	0.39	-0.99 ± 0.19	-0.09 ± 0.28	-	-
4	0.38	-	-	-1.34 ± 0.23 -0.31 ± 0.26	-
5	0.41	-	-	-	-0.81 ± 0.16
6	0.38	-	-	-0.98 ± 0.49 0.02 ± 0.49	-0.33 ± 0.41

*u*: infinitesimal genetic effect and associated heritability (*h^2^*)

q_a_: additive qtl effect

q_d_: dominance qtl effect

q_a_(sex): additive qtl effect nested by sex, males and females

snp: snp additive effect (SNP number 5)

**Figure 3:**
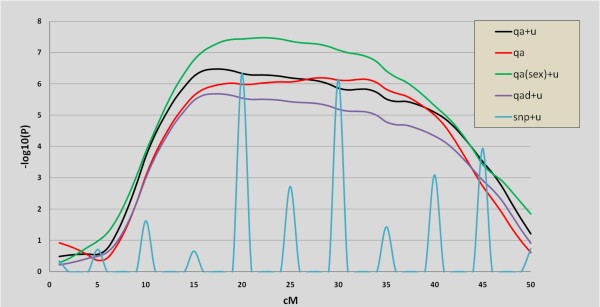
**P-value profiles with different models for carcass length in an Iberian × Landrace intercross**.

EFFECT

u cross 1 pedigree pedigreefile

where cross indicates that this is a cross classified variable (as opposed to a covariate) and 1 is the position of the variable(the individual); pedigree is a reserved word specifying the covariance matrix of the random variable; diagonal or user specified covariance matrices can be used as well. The comparison between both profiles is in Figure 3 in red (no infinitesimal effect) and black lines (infinitesimal effect). Two interesting remarks can be made. The first one is that the optimum position is shifted from 28 cM to 17 cM without and with the infinitesimal effect, respectively. The second one is that the model with the infinitesimal effect slightly increases power because the QTL is more significant when an infinitesimal effect is fitted (Table 3). Although the infinitesimal effect in crosses should be interpreted with care when the genetic architecture in each founder population is very different, our experience shows that, whenever possible, introducing an infinitesimal effect decreases the rate of false positives enormously.

**Table 3:** Likelihood ratio tests between alternative models from Table 2

Test	Meaning	P-value
1 vs. only fixed effects	QTL additive effect	6 × 10^-7^
2 vs. 0	QTL additive effect conditional on infinitesimal effect	3 × 10^-7^
3 vs. 0	QTL additive and dominance effects conditional on infinitesimal effect	2 × 10^-6^
4 vs. 0	QTL additive effect nested within sex conditional on infinitesimal effect	3 × 10^-8^
5 vs. 0	Raw association signal conditional on infinitesimal effect	5 × 10^-7^
6 vs. 4	Association (SNP) signal conditional on linkage (QTL) signal	0.43
6 vs. 5	Linkage signal conditional on SNP signal	7 × 10^-3^

Next we tested whether the QTL behaved additively or not. To do that, we optionally fitted a dominance effect. In Qxpak notation,

QTL

qtl_add fix_a global

qtl_dom fix_ad global

where global means that the QTL is fitted throughout all positions. Qxpak allows us to fit an only dominant effect QTL using fix_d word. The resulting P value profiles are in red (additive) and magenta (additive and dominant). Clearly, the QTL behaves in an additive manner  = -0.99 ± 0.19 and *d *= -0.09 ± 0.28. However, the most surprising result, which was not tested in the original manuscript, is that there is a strong sex × QTL interaction (model 4). It is clearly observed that the QTL additive effect affects males only (Table 2 and 3). This is also observed in the P value profile (the green line in Figure 3). In Qxpak, the interaction between covariates and cross classified effects, say sex, is denoted as

QTL

qtl_add_sex fix_a global (sex)

To finish the analyses, we also fitted every SNP separately as in an association study (cyan line in Figure 3). SNP 5 is found to be the most strongly associated with the trait. A main problem in these kind of analyses is that linkage and linkage disequilibrium signals are confounded, especially when the allele marker frequencies are different between founder populations, which is a common observation if these are distant genetically. To further explore this issue, we fitted a complete model that includes both the additive QTL and SNP 5 (model 6, table 2) and we tested it against a model with only linkage (model 4) or only association signals (model 5). The tests (Table 3) suggest that most of the signal comes from linkage rather than population disequilibrium because the SNP is not significant once the QTL is fitted whereas the opposite is not true: the QTL is significant even after fitting the SNP. Therefore, one can conclude that there is a locus on SSC4 that affects males only and is related to development. Note that the same procedure can be used to determine how many loci are significant: once a first significant locus is fitted - be it a SNP or a QTL - it can be tested whether a new locus is significant conditional on the first one.

## Conclusion

Qxpak.5 provides a flexible tool for genomics analysis that can be used in a wide variety of situations with ample genetic and statistical modeling flexibility while implementing a coherent, unified mixed model approach. The next step is to deal with complete genome sequence data for association studies. To that end, we believe the principles behind the use of the molecular coancestry matrix in GWAS should be a promising starting point.

## Availability and requirements

Qxpak is free with no restriction on its use; 64 bit linux, DOS and cygwin executables, manual and examples are in additional file or at http://www.icrea.cat/Web/OtherSectionViewer.aspx?key=485&titol=Software:%20Qxpak&researcher=255

## List of abbreviations used

GWAS: genome wide association study; IBD: identity by descent; MM: mixed model; QTL: quantitative trait locus; SNP: single nucleotide polymorphism.

## Authors' contributions

MPE conceived and carried out the research. IM provided key software code and guidelines. MPE wrote the manuscript with IM's assistance. All authors read and approved the final manuscript.

## Supplementary Material

Additional file 1 **Binary code with manual and examples**. zipped file containing complete manual, examples and binaries for linux 64 bits, DOS and cygwin. Click here for file
