# Supplementary material for: Qxpak.5: Old mixed model solutions for new genomics problems
Source: BMC Bioinformatics. 2011 May 25;12:202. doi: 10.1186/1471-2105-12-202 (PMC3123239; doi:10.1186/1471-2105-12-202)
Supplement: Additional file 1 — Binary code with manual and examples. zipped file containing complete manual, examples and binaries for linux 64 bits, DOS and cygwin. [file 1471-2105-12-202-S1.ZIP › Qxpak.5.03_readme_BI.pdf]

Qxpak.5

Old mixed model solutions for new genomics problems

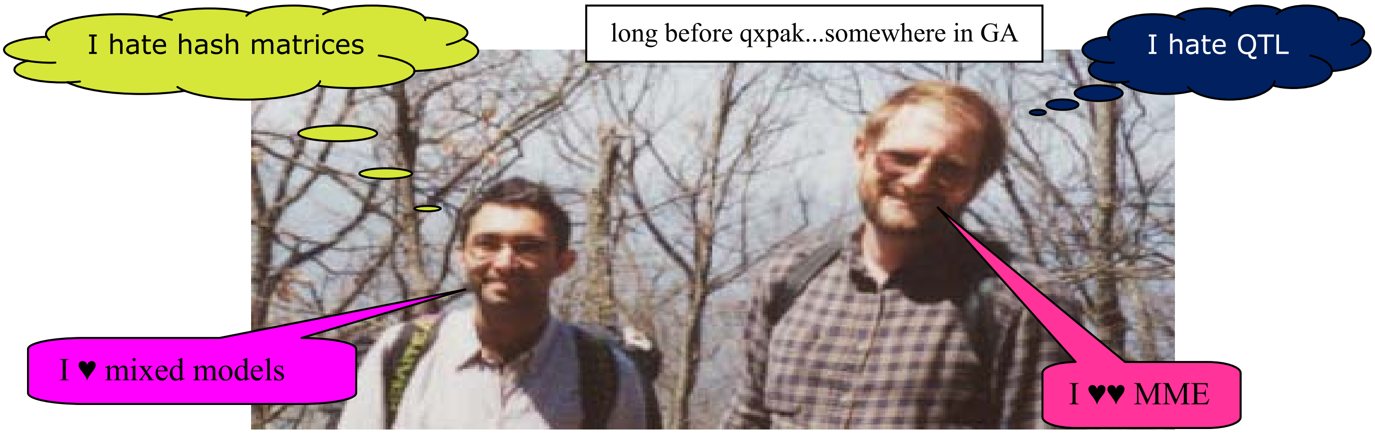

by

Miguel Pérez-Enciso[1,2] & Ignacy Misztal[3]

[1] Institut Catalá de Reçerca i Estudis Avançats (ICREA), 08010 Barcelona, SPAIN
http://www.icrea.cat/Web/ScientificForm.aspx?key=255

[2] Department of Food and Animal Science, Universitat Autònoma de Barcelona, 08193 Bellaterra, SPAIN

[3] Department of Animal and Dairy Science, University of Georgia, Athens, GA 30602, USA
http://nce.ads.uga.edu/~ignacy/

if (comments | suggestions) {miguel.perez@uab.es};

INTRODUCTION

MOTIVATION

This software aims at simplifying statistical genetic analyses implementing a coherent and unified mixed model approach. The goal is to provide software that can be used in a wide variety of situations with ample genetic and statistical modeling flexibility.

WHAT IS IT?

QxPak is a fortran95 (sorry no C or C++ nor R) package for extremely versatile statistical genomics and quantitative trait loci (QTL) and association analyses. Further, it accommodates the type of problems faced in genetical genomics, i.e., a huge number of sequential analyses where each mRNA level is treated as a different trait. Finally, it offers an extremely flexible modeling of linkage (QTL) analyses, or mixed linkage – association analyses, imprinting, sex linked... The new version (v. 5) allows us to include IBD matrices obtained with molecular information, or user defined matrices that can be read from an input file. Either the direct or the inverse matrices can be used as input. This feature can be used for genome selection (although is not optimum from a computational point of view) or – more importantly – to correct for population structure in association studies. In crosses, it can accommodate more than two parental lines, which are not necessarily assumed to be inbred.

Not competing but rather complementary to other popular packages like Plink or GenABel, this software offers a wide statistical modeling flexibility, e.g., multivariate models where these can differ from trait to trait, combining different IBD matrices, including pedigree and marker based matrices, or importantly allowing for any pattern of missing data. The package takes advantage of the well developed statistical theory for mixed models together with efficient algorithms for obtaining (restricted) maximum likelihood estimates. Last, classical features like BLUP estimation, REML variance estimations are accommodated due to package inheritance.

Parameter estimates are obtained using Maximum Likelihood or REML via an EM algorithm as programmed by Ignacy Misztal. The specialized package for sparse matrices FSPAK is used and QTL mixed model programs.

AVAILABILITY

This program is free. At the time of writing, the software is available at http://www.icrea.cat/Web/ScientificForm.aspx?key=255; click in 'Other sections'.

PLEASE CITE US

PÉREZ-ENCISO, M., and I. MISZTAL, 2004 Qxpak: a versatile mixed model application for genetical genomics and QTL analyses. Bioinformatics **20:** 2792-2798.

PÉREZ-ENCISO, M., and I. MISZTAL, (????) Qxpak5: Old mixed model solutions for new genomics problems. To be submitted.

BUT DONT TRUST US (BLINDLY)

Note that the authors can not be liable for any result of this program, and that there is no warranty that the program is error free. Comments on bugs are certainly welcome: miguel.perez@uab.es.

INSTALLING QXPAK

Compiled 64 bit linux executables are currently distributed. Download it into the directory, Probably you have to make it executable with the command

```
chmod +x qxpak
```

To run it, type

```
./qxpak
```

The program then asks for the name of the parameter file. If you prefer you can type

```
echo parameter.file | ./qxpak
```

where *parameter.file* is the name of your parameter file, and the parameter file is read automatically. You should also find a folder with examples. If you experience some weird problems like not reading properly the files or crashing unexpectedly, it might be that the format of the files is MS DOS rather than linux, try `dos2unix` command, e.g.,

```
dos2unix qxpak.input.files
```

IS QXPAK FOR YOU?

If you are already familiar with Qxpak, the only new thing you need to learn in v.5 is about including the molecular IBD matrix. Let us nonetheless recapitulate on the main kind of analyses you may use Qxpak for:

1. **Regular mixed model solving:** The package can be used as a the original Misztal's programas for usual REML analyses. Some modeling flexibility has been lost, however, as maternal effects has not been implemented. To compensate, a more friendly parameter file can be used. A typical application is to estimate heritabilities and genetic correlations.

2. **QTL** (multitrait, multiQTL, etc): The initial goal of QxPak was to carry out linkage (QTL) analyses. MultiQTL and multitrait models have been fully implemented. As detailed below, a wide range of genetic modeling options are possible.

3. **Segment analysis**. As proposed elsewhere, an alternative to classical QTL scans is to assess the fraction of genetic variance explained by a set of chromosome segments. The method consists of partitioning the genome in a series of segments, delimiting specific genome regions that we are interested in analyzing. To an extent,

4. **Multitrait** (Microarray): In the context of genetical genomics analyses, each mRNA level is treated as a different trait and, thus, a large number of univariate QTL analysis is required. QxPak provides an automated option to allow for this. The limitation is that the same model is applied to all mRNA traits, but otherwise modeling is equally flexible.

5. **Association:** Given the wide availability of SNPs, one may also be interested in testing the effect of each one in turn, as in a repeated association study like a usual genome scan (WGAS). QxPak allows defining a QTL as SNP with starting and end positions. The `snp_` option allows including a dominant effect but can be applied to biallelic markers only, the `ld_` option can be applied to multiallelic markers but no dominance can be included. For multiallelic markers, an option is to treat them as random (option `ld_ran`) instead of estimating an effect for every allele (fixed option `ld_fix`). The user may wish to use `MEMORY_RAM = yes` and `TRANSPOSE` options (see below) for large scale genotyping data.

6. **Molecular relationship matrix:** This is the main new option in Qxpak5. The possibility of including random correlated genetic effects is of utmost importance to correct for structure correction and minimizing the risk of false positives in large scale association analyses, as it is for genomic selection procedures. Qxpak allows for the possibility of including any number of user defined matrices (that can be either the direct or the inverse matrix), or allow Qxpak to compute a number of marker based relationship matrices.

7. **Sequence based association studies:** In the immediate future, we expect to have complete or near complete genomes in populations making it possible to carry out true complete genome wide association studies. Although Qxpak is not ready yet for that challenge, we anticipate that polymorphism based matrices to quantify genetic relationship will be a key instrument. Qxpak offers some options that may be useful, like scans on

marker based relationship matrices computed at predetermined positions. If desired, this can be corrected for a genome wide relationship matrix.

A fundamental characteristic of Qxpak is that the modeling options for the QTL can be combined almost ad libitum, for instance, one could do a SNP scan with each of a defined number of traits and can include an additional QTL modelled in the classical way. Or can we include a genome wide / chromosome wide molecular relationship matrix. All in all a wide variety of analyses is possible. Some QTL can be scanned and some can be defined as segment. We strongly recommend combining QTL analysis, be it linkage or association, with an infinitesimal effect.

IF NOT

Play the official Qxpak5 song: You can't always get what you want.

MAIN FEATURES

- ✓ Multitrait.
- ✓ Linkage and/or association tests.
- ✓ Genetic effects can be modeled as fixed effects or random with covariance matrices computed based on marker and pedigree or only marker information.
- ✓ Genomic relationship matrices based on molecular information can be included
- ✓ Multi QTL.
- ✓ Different models per trait, including different QTL modeling by trait.
- ✓ Any number of chromosomes can be analyzed jointly.
- ✓ Missing observations.
- ✓ (approximate) Dealing with missing markers.
- ✓ Flexible QTL modeling.
- ✓ Custom epistasis modeling
- ✓ Custom defined IBD or covariance matrices.
- ✓ Sex chromosome modeling.
- ✓ Imprinting
- ✓ Allows for multibreed (n>2) crosses
- ✓ Chromosome segment modeling.
- ✓ QTL x other class effect (say sex) interaction.
- ✓ Linkage vs Pleiotropy tests.
- ✓ Fast – sparse matrix based - computing strategies.
- ✓ Can also be used efficiently for classical (infinitesimal model) analyses.
- ✓ All individuals are included in the analyses, irrespective of whether they are F2, backcross, F1, or purebred, or a mixture of origins.
- ✓ Provides errors of estimates, except of variance components.

CAUTIONS AND TIPS

Qxpak is an extremely flexible package that allows a very large number of modeling options. Because of that, though, the user is cautioned that the interpretation of the results may not be always straighforward. Here are some recommendations and warnings:

- Qxpak simply disregards untyped markers and thus may result in inaccurate IBD probabilities if many markers are missing, specially if the first individuals in the pedigree are missing.
- Qxpak checks marker consistency for the `fix_` and `ran_` options but not with the `snp_` or `ld_` options.
- If you are carrying out a large GWAS, use the MEMORY_RAM option (see parameter file doc), use also the transpose option for marker file. In any case you should not expect an ultrafast performance as in Plink. But you can include complex models like multitrait or the infinitesimal effect.
- Some models may result in numerically unstable computations that can yield bizarre results. This applies particularly to random qtl effects.
- It is generally useless to try to fit too complicated models with small datasets.
- Adding extra parameters diminishes power. For instance, if a dominance effect is not significant, use the **fix_a** option rather than **fix_ad**.
- Do not include more than two QTL initially, as this increases hugely the number of analyses to be carried out.
- Modeling the QTL as random (mix or ran options) increases geometrically the CPU time in obtaining the IBD probabilities. Besides, these analyses can be prone to numeric problems with small datasets, low informativity markers or if too many traits or several QTL are being fitted.
- The segment analysis can be a useful starting strategy to browse the genome with a minimum CPU expense.

WARNING: IMPORTANT FORMATTING ISSUES

- Separators between fields must be spaces, tabs may not work.
- Missing values are specified by 0 for markers, phenotypes or effects. This may create some unexpected results if a covariate has 0 as true value, as that observation will be deleted from the analysis. The user should set a true 0 to the value 0.000001 EXACTLY , otherwise that record will be deleted from the analysis.
- The first field in the pedigree file, data file and marker file must be the individual.
- If you experience some weird problems like not reading properly the files or crashing unexpectedly, it might be that the format of the files is MS DOS rather than linux, try dos2unix command. Try also to remove the z* files and restart the program.
- Marker alleles are specified by integer numbers, for SNPs, the alleles have to be 1 and 2.
- In pedigree file, heterogametic sex individuals are coded as 1 and homogametic as 2. In mammals, males are 1 and females, 2.
- In pedigree file, parents must precede any offspring, alphanumeric characters are accepted for individual codes.
- Comments in parameter file can be included at the beginning or end of lines with # or !.
- We recommed to use only upper or lower case, and not a mixture, to name chromosomes and individuals.

USER'S GUIDE

Qxpak requires normally four files: a parameter file with input details, a data file containing phenotypes and any effect that may be included in the model, a pedigree file and a marker file with genotypes. Not all included effects in the data file need to be included in the model, i.e., the same files can be used to run a diversity of analyses. The marker file is not required if the program is used without QTL (e.g., to obtain REML or ML estimates in an infinitesimal model). In an association analysis, the pedigree file may not be required, although a dummy file is created.

Other input files that can be required are user defined covariance matrices (see options `g_usr` and `g_usr_inv` below): a particular covariance matrix can be specified with a file, or haplotype files, which specify known phases other than deduced from marker info. If not provided, the program automatically detects which phases are known with certitude and which not.

PARAMETER FILE

The parameter file is separated in sections (in upper case here for clarity). In general, the sections can be in any order, although some restrictions apply. Most of sections are also optional, with default values as provided. Main sections are:

`ML_OPTION (Optional, Values: Y/N, Default: Y)`

Specifies whether Maximum likelihood (Yes) or Restricted Maximum Likelihood (No) is used. Yes, Y, yes or y are equivalent.

`MEMORY_RAM (Optional, Values: Y/N, Default: N)`

Specifies whether all data and markers are loaded in RAM rather than reading and writing files. It works only when QTL is defined as snp or ld (see below). It does not work if multitrait option is defined.

`MULTITRAIT_OPTION (Optional, Values: Y/N, Default: N, single trait)`

If Yes, the next field must be the number of traits analyzed sequentially.

Example 1: (12300 sequential trait analyses)

y 12300

`DATAFILE (Optional, Default: qxpak.dat)`

Specifies name of file contaning individual, records, and effects. The first record must contain the individual.

`OUTFILE (Optional, Default: qxpak.out)`

If the keyword `append` is present, new analyses are appended to existing file

```
qxpak.out             !every new analysis overwrites the file
qxpak.out.all append !output appended
```

`PEDIGREEFILE (Optional, Default: qxpak.ped)`

File format detailed below.

`MARKERFILE (Optional, Default: qxpak.mkr)`

Specifies name of file contaning marker information. If optional keyword **transpose** is written after marker file, marker file can be written where snps are arranged by rows rather than by oclumns. The format of the file is detailed below.

<u>Examples</u>

qxpak.marker

qxpak.marker transpose

qxpak.mkr.t   t   !transpose can be abbreviated by t or T

`HAPLOTYPEFILE (Optional, Default: no haplotypes read)`
Specifies the name of file contaning the haplotypes known. (details below).

`SUMMARYFILE (Optional, Default: none)`
Used only with MULTITRAIT_OPTION on. The format of the file is detailed below.

`MARKER_POSITIONS (Obligatory if QTL included)`
Format
name_of_chromosome, positions of each marker in cM (real)

or

name_of_chromosome, -number_of_markers (, spacing_in_cM)

- The number of chromosomes is identified by the number of lines of this section
- Comments can be included within the section with # sign
- The program detects automatically the number of markers by counting the number of positions, which should match the number of markers in the marker file, otherwise the program may stop.
- Marker positions must be entered in order
- If the number of markers in the marker file does not match the number of positions in this section, the program discards unspecified markers.

Example 1:
```
chr77 0. 10.13 20.1
#chr13 1 2 23.      !LINE IGNORED
```

```
Example 2:
chr77 -100          !100 markers spaced every cM
chr13 -2000 .05     !2000 markers spaced every 0.05 cM
```

```
Example 3: WRONG! markers not in order. crash!
chr77 0. 20.1 10.13
```

Note: spacing between markers do not matter for association analysis.

`SEX_CHROMOSOME (Obligatory if sex chr included)` `NEW!`
This section, if present, specifies which is the sex chromosome; optionally, a real number specifies the length of the pseudoautosomal region (PAR). If not present, the whole chromosome is assumed to be non-recombining region (NRY). The method described (Pérez-Enciso *et al.* 2002) is applied assuming a dosage compensation parameter ψ=0.5. In PAR, dosage compensation does not apply. The program identifies automatically which markers are pseudoautosomal (position smaller than male chr length) and which are not (position larger male chr length).

- It is known that PAR are in both telomeric regions, at least in humans, Qxpak accounts for only one region.
- In species where the heterogametic sex is the female, females should be coded as 1 in the pedigree file.
- Length of PAR should be smaller than the total length of X chromosome (specified in `MARKER_POSITIONS` section).
- Title can be abbreviated as `SEX_CHR`

```
Examples:
chrx     !the whole chr is NRY
chrw 10  !all markers in position ≤ 10 cM assumed to be pseudoautosomal
chrw -10 !wrong
```

`QTL (Obligatory if any marker effect included)`

Declares genetic effects to be included in the model (in section TRAIT below). Not all QTL declared here must be included in the model, but it is recommended to specify the minimum number of loci required to minimize computing. The number of QTL is identified by the number of lines of this section. Comments can be included within the section with # or ! signs (do not count as QTL). The format is

QTL_name, QTL_type, (segment), Position_specification, (Nested) (Imprinting paternal) (Imprinting maternal)

Qtl types defined are (see advanced theory below):

- `fix_a:` additive fixed effect
- `fix_d:` dominant fixed effect
- `fix_ad:` add+dom fixed effect
- `snp_a:` additive fixed effect (SNP)
- `snp_d:` dominant fixed effect (SNP)
- `snp_ad:` add+dom fixed effect (SNP)
- `ld_fix:` additive association study (multiallelic marker)
- `ld_ran:` additive association study (multiallelic marker)
- `ran_1:` additive random effect (common variance to all breeds)
- `ran_2:` additive random effects (different variance per breed)
- `ran_01:` additive random effects (variance for breed 1 is set to zero)
- `ran_10:` additive random effects (variance for breed 2 is set to zero)
- `ran_mol:` additive random effects with marker based variance (NEW!)
- `mix_1a:` mixed effect (fix_a + ran_1)
- `mix_01a:` mixed effect (fix_a + ran_01)
- `mix_10a:` mixed effect (fix_a + ran_10)
- `mix_1d:` mixed effect (fix_d + ran_1)
- `mix_01d:` mixed effect (fix_d + ran_01)
- `mix_10d:` mixed effect (fix_d + ran_10)
- `mix_1ad:` mixed effect (fix_ad + ran_1)
- `mix_01ad:` mixed effect (fix_ad + ran_01)
- `mix_10ad:` mixed effect (fix_ad + ran_10)
- `mix_2a:` mixed effect (fix_a + ran_2)
- `mix_2d:` mixed effect (fix_d + ran_2)
- `mix_2ad:` mixed effect (fix_ad + ran_2)
- `epi_fix:` epistasis between fix QTL
- `epi_ran:` epistasis between ran QTL
- `epi_snp:` epistasis between snp QTL

Position specification is given according to three formats in all cases except `ran_mol`

- `chr_name, start_position, end_position`: repeated for many chrs as needed, example:

```
qtl1 fix_a chr1 0 100. chr12  10.  12.1
snp1 snp_ad chr1 1 100 chr12 10 12
```

for regular QTL, the positions specifies the starting and end chromosome positions (in cM) where the QTL is scanned; for SNPs, it specifies the first and last markers to be considered. For a SNP (snp_ or ld_), it means that first 100 markers in chr1 and markers 10, 11 and 12 are fitted one at a time in the model.

- `chr_name, all`: all positions are scanned for that chr for that QTL, example:

```
qtl1 fix_a chr7 all
```

  or a combination

```
qtl1 fix_a chr1 all chr12 0. 13
```

- `global`: means all positions (markers) in all chrs are scanned for that QTL, example:

```
qtl2 fix_a global
mkr2 ld_fix global
```

For **ran_mol modeling**, the above options imply that a relationship matrix is computed for all markers comprised within the bounds defined. For instance

```
qms ran_mol chr1 all chr12 1 13
```

means that a random genetic effect is defined with relationship matrix computed using all markers in chr1 and markers 1 through 13 of chr12. Similarly

```
qms ran_mol global
```

means that the relationship matrix is computed with all markers. In this case, the global label accepts two modifiers, one is 'chr' and the second one is the step width, an integer number.

```
qms ran_mol global chr
```

means that different relationship matrices are computed by chromosome, as in a chromosome by chromosome scan. The option

```
qms ran_mol global 100
```

means that consecutive relationship matrices every 100 markers are computed. Note: if a chromosome harbors more markers than an exact multiple of `step`, the remainder markers are assigned to the previous matrix, i.e., the number of relationship matrices computed are int(number_of_markers / step).

If you want a particular QTL(s) be **nested** within a class effect, say sex, you specify (name_of_effect) after or before specifying scan positions. Effect must be defined in the EFFECT section and must be defined as `cross` (class effect, see below). As of version 5.03, you can specify nested separately for additive or dominant components by typing `nested_add` or `nested_dom` after the parentheses. This applies only to `fix_ad` and `mix_*ad` qtl types.

A QTL is defined as **segment** typing `segment` (`seg` for short) after the QTL definition. In a segment locus, the whole fragment between beginning and end positions are considered rather than in a scan, where 1 cM positions are analyzed.

Imprinting is modelled with keywords `imprinting maternal` or `imprinting paternal`, which can be shortened as `imp, mat and pat`, respectively.

Example:
```
qt1  fix_ad (sex)  chr1 0 3
qtl2 mix_1ad  chr2 all chr1 4. 13.2
q3sg  fix_a  segment global (sex)       # segment qtl nested within sex
ia3  fix_ad  global (sex) nested_add    # qtl nested within sex applies only to
                                         # additive component
m4all  mix_1ad  global (batch) nested_dom  # qtl nested within batch in dominant
                                            # component only
IGF2 snp_a global imp maternal
```

```
DECR snp_ad chr4 10 20
qseg fix_ad seg chr4 11 23
```

Here QTL q3sg is defined as segment, global, and sex specific. This means that the average probability across the whole genome of being of origin A or B is computed. That is the terms $P(g_{ik}^1 \in U, g_{ik}^2 \in W)$ corresponding to the whole genome are included in the model, and that a separate regressioon is carried out within each sex. As for QTL qseg, the relevant probabilities are obtained between chr4 positions 11 and 23 cM only. IGF2 is modelled with maternal imprinting.

There is a slight variant to model **epistatic QTL**, the format is

QTL_name, QTL_type, QTL_1, QTL_2, (`custom`, weights 1 through 9)

Segment and imprinting does **not** apply. Nested option does apply (**not tested!**). You do not need to define positions again, the program scans all those for which QTL_1 and QTL_2 are defined. QTL_1 and QTL_2 **must** have been defined previously, even if they are not used in the model. The reserved word **`custom`** allows the user to set its own desired epistatic coefficients, applicable only to epi_fix or epi_snp options. If you write custom, the program expects 9 integer positive or negative numbers. These numbers indicate which haplotypes are to be pooled, the numbers are read in order AABB AABb AAbb AaBB AaBb Aabb aaBB aaBb aabb, where *A* and *B* are the two loci. Equal numbers mean that these haplotypes are added up. For instance, the classical axa, axd, dxa and dxd coefficients are

```
1 2 -1 3 4 -3 -1 -2 1
```

with four coefficients. In the example below

```
1 2 -1 1 2 -1 1 1 1
```

Two regression or haplotype pools are computed.

p(AABB) - p(AAbb) + p(AaBB) - p(Aabb) + p(aaBB) + p(aaBb) + p(aabb), and

p(AABb) + p(AaBb)

Negative weights apply only to fix option. The following weights in a SNP option

```
1 2 1 1 2 1 1 1 1
```

means that two effects are estimated with pooled genotypes AABb and AaBb (level 2) and the rest (level 1)

More examples

```
q01 epi_fix q0 q1      !ok
q34 epi_snp q3 q4      !ok
q567 epi_ran q5 q6 q7  !ok, but think what you do
q18 epi_fix q1 q8      !WRONG: q8 not defined
q18 epi_fix q1 q3      !WRONG: either q1 or q3 are not fix
q12 epi_fix q1 q2      !WRONG: epistasis does not apply to mix qtl
q01 epi_fix q0 q1  custom 1 2 -1 1 2 -1 1 1 1 !ok
q34 epi_snp q3 q4  custom 1 2 -1 1 2 -1 1 1 1 !WRONG negative coeffns and snp
q01 epi_fix q0 q1  custom 1 2 -1 1 2 -1 1 1   !WRONG: a number is missing
```

Warning

- Nested option works only with `fix_*` and `mix_*` types.
- If you define a SNP to be scanned between markers 10 and 20, say, and any marker in between is not biallelic, the program will STOP. Use `LD_` option instead.

- Option segment is incompatible with SNP_ or LD_ type.
- It should be noted that the likelihood under the null hypothesis must be recalculated with every new SNP if there are missing genotypes. Thus, it is discouraged to define SNPs that are not actually used in the model, as a full positions' scan is done for both the null and alternative hypotheses.
- The imprinting option has not been fully tested.
- Models with qtl defined as random tend to be unstable, with sometimes bizarre log profiles.

STANDARDIZED_MOL (Optional, Values: Y/N, Default: N)
Specifies whether the molecular relationship matrix is standardized or not. Briefly (see theory below) it means whether all SNPs are given the same weight (standardized=Yes) or not. Yes, Y, yes or y are equivalent. Applies only if any genetic effect is defined as ran_mol, and is applied to all ran_mol effects.

EFFECT (Obligatory)
Specifies the number and kind of effects that may be included in the model; as with QTL, not all effects need to be included in the model but is good to keep the section as parsimonious as possible. Comments can be included within the section with # sign, that effect is not considered. The format is

Name_of_effect, effect type, position_in_datafile, (random_type (random_file_name))

The effecttype can be cross (crossclassified) or cov (covariate). By default, everything is fixed, Random effects are specified with random_type, which can take any of these keywords (see Misztal's programs documentation):

- diagonal: covariance matrix is diagonal
- pedigree: infinitesimal, additive animal model, pedigree file required (deprecated *add_animal*)
- g_usr_inv: user defined matrix, to be inverted, random file name required,
- g_usr: user defined matrix, inverse provided, random file name required

Different effects can be located in the same data file position, this can be useful, e.g., if one wants to have a covariate nested for one trait, but not nested for another trait. Also, an effect for one trait can be jointly analyzed as a trait, for instance, one can analyze bivariate analysis of weight and age, where age is covariate for weight.

Example:
```
sex cross 2
litter cross 6 diagonal
Age cov    3
Weight  cov   3 (sex)
Grasa cov   4
u0  cross 1 pedigree qxpak.ped      !infinitesimal anim effect
gmkr  cross 1 g_usr  zmkr.inverse   !inverse IBD matrix provided
```

Note that the same column can be defined in two different manners, as above for position 1. **Note, QxPak writes the inverse of the molecular relationship matrix with names zmkr.i.pos, where i is chromosome and pos, segment** so these can be used in later analyses without repeating the whole procedure of defining a qtl as ran_mol and computing the zmkr* files again.

TRAIT (Obligatory)
Defines model for each trait. The number of traits is identified by the number of lines in this section. Comments can be included within the section with # sign. A missing trait is specified by 0. If MULTITRAIT option is true, then the trait position refers to that of first trait, trait 2 is supposed to be in trait position plus 1, etc. See example 3 below.

Trait_name position_in_data_file name of effects affecting the trait, including qtls

Note that the same column can be defined in two different manners. The same values can be treated as a trait or as a covariate for the next trait. This flexibility is inherited directly from Misztal's programs.

Example:

```
Age_trait   3  qtl2 e1  sex
fat       15 u0   sex Age
Epitrait   15 u0   sex Age q01        !You can fit 1 epistatic qtl only
Epitrait1   15 u0  sex Age q1 q01     !or one
Epitrait2   15 u0  sex Age q0 q1 q01  !or both
#Effect_ignored 12 u0 f3 wr
```

The above example would fit five traits simultaneously! If you want single trait analysis, you should comment the rest of traits

```
Age_trait   3  qtl2 e1  sex
!fat       15 u0   sex Age
!Epitrait   15 u0   sex Age q01        !You can fit 1 epistatic qtl only
!Epitrait1   15 u0  sex Age q1 q01     !or one
!Epitrait2   15 u0  sex Age q0 q1 q01  !or both
#Effect_ignored 12 u0 f3 wr
```

TEST (Optional)

Specifies which are the effects deleted to make the null model; effects can be tested separately for each trait. There are three basic options

`name_of_effect` OR `name_of_qtl` : removes the effect for all traits in the null model
`name_of_qtl qtl_type` : keeps the QTL of type qtl_type in the null model for all traits
`name_of_trait name_of_effect` : removes the effect for that trait

Several effects can be in the same line. In the case of QTL, the user can specify submodels. For instance, if the QTL is specified as `fix_ad`, the dominance effect can be tested by specifying `fix_a`. Tests are effective only if the models are hierarchised, eg, `ran_1` cannot be tested against `fix_a`, but `mix_1ad` can be tested against `ran_1` or `fix_a` or `fix_ad`.

```
QTL
qtl2 fix_ad global
TRAIT
trait1 mean qtl2 sex
trait2 mean qtl2 sex
...
```

```
Example 1:
TEST
qtl2              !removes qtl2 for both traits
```

```
Example 2:
TEST
qtl2 sex          !deletes qtl2 and sex effects for both traits
```

```
Example 3:
TEST
qtl2 fix_a        !qtl2 is redefined as fix_a in the null model for both traits
```

```
Example 4:
TEST
trait1 qtl2 fix_a trait2 sex !you guess
```

```
# Example 1: qxpak_1.par
# Simple F2 cross between
# inbred lines
ML_OPTION
Y
DATAFILE
qxpak_1.dat
OUTFILE
qxpak_1.out
PEDIGREEFILE
qxpak_1.ped
MARKERFILE
qxpak_1.mkr
MARKER_POSITIONS
!--> equivalent to chr1 -5
10.
chr1 0.0 10 20 30 40
QTL  !--> no dominance
qtl_1 fix_a  chr1 all
EFFECT
mean cross 2
age covariate 3
TRAIT
t1 5 mean qtl_1
TEST !--> tests QTL effect
qtl_1
```

```
# Example 2: large scale
# association study correcting
#  by structure with IBD matrix
ML_OPTION
Y
DATAFILE
gwas.dat
OUTFILE
gwas.out
PEDIGREEFILE ! not needed
gwas.ped
MARKERFILE
gwas.mkr transpose
MARKER_POSITIONS
chr1 -10000
QTL
snp snp_ad global
gmkr ran_mol global
EFFECT
mean cross 2
sex cross 13
TRAIT
t1 5 mean sex snp gmkr
TEST !--> tests SNP effect
snp
```

DATA FILE

It is a free format file that contains the records and the effects, it can contain more traits and more effects than actually used in the model.

- ✓ Individual id MUST be the first column, and can be numeric or alphanumeric. Individual records can be entered in any order, i.e., they do not need to be the same as in marker or pedigree file.
- ✓ Missing values in the trait or effects are indicated by 0, this has no effect for covariates. This may create some unexpected results if a covariate has 0 as true value, as that observation will be deleted from the analysis. The user should set the 0 to the value 0.000001 EXACTLY if that number is to be taken as true 0.

Example :
```
1a    2 3.4 2.7 -14    ! individual 1 record
ind21 1 1.4 2.3  4     ! individual 21 record
13    1 2.  3.  0      ! missing information for trait in column 5
```

PEDIGREE FILE

The format is

`individual, father, mother, ((sex), breed)`

Sex and breed fields are optional if analyzing non sex chromosomes and within breed populations. Otherwise they are obligatory.

- ✓ Individual, father and mother can be numeric or alphanumeric. Individuals need not be coded.

- ✓ Unknown parents are specified with code 0.
- ✓ Breed indicates the genetic origin of the individual for parentals in crosses between lines. Breed can take integer values starting with 1, 2... if only 1 is specified, then it is assumed to be a common variance for that qtl. If all individuals pertain to the same breed, the same code should be used for all founders.
- ✓ In modeling the QTL, the fixed options are not allowed if only one breed is specified.
- ✓ With random options, suffix '_1' (ran_1, mix_1a, ...) indicates that the genetic variance is the same in both founder breeds. The '_2' suffix forces a genetic variance for breeds 1 and 2 to be estimated separately.
- ✓ Breed code is effective only for those individuals with at least one parent unknown. Otherwise, the genetic origin is traced using the pedigree and marker information.
- ✓ A pedigree file is required for QTL analysis, if none is specified, a dummy file is created assuming all individuals are unrelated.

Cautions

- ❖ The last field (breed) can be missing, then, all individuals are assumed to be from the same breed.
- ❖ Both sex and breed fields can be missing. No sex chrs. assumed in this case.
- ❖ Breed cannot be present when sex is absent.
- ❖ If you define a single breed, modeling options fix_ or mix_ cannot be used.
- ❖ Breed field is relevant only for those indivs where you have at least one parent missing. If parents, are known the program automatically computes the breed composition given marker information. In the example below, it does not matter whether full sibs 3, 4, or 5 are assigned to breeds 1 or 2; but it does for the first two individuals, 1 and 2.

```
Example 1: !parents for
inds 1 & 2 are unknown
1  0  0 2 1
2a 0  0 1 2
3  2a 1 1 2
4  2  1 1 2
5  4  3 1 1
```

```
Example 2: It is
assumed that all indivs
are from one breed
(field breed missing)
1  0  0 2
2a 0  0 1
3  2a 1 1
4  2  1 2
[illegible]
```

```
Example 3: It is assumed that all indivs are from one breed
and that no sex chr is analyzed (program crashes otherwise)
1 0 0
2 0 0
3 2 1
4 2 1
5 4 3

Example 4: !three breeds detected
1 0 0 2 1
2 0 0 1 2
3 0 0 1 3
4 2 1 1 2
5 4 3 1 1
```

MARKER FILE

There are TWO main options, the usual format and the transposed format.

Usual Format:

For each chromosome,

first record: chromosome name

successive records: individual, allele1_mkr1, allele2_mkr1, etc

- All chrs can be specified in a single file, even if the QTL is not scanned throughout, the program identifies which IBD probabilities and what positions need to be computed.
- Missing alleles are specified by 0.
- IBD probs are saved in a file with name z*, these files should not be deleted except if one wants to have IBD probabilities recomputed.
- In males X chromosome, missing alleles must labelled as 0.
- In sex chromosomes, individuals labelled as males cannot have two alleles in the non pseudoautosomal region. The program crashes!

Transposed format

It is appropriate when many more markers than individuals are available. The format is:

First row: list of individual codes

Successive rows: SNP_name, chr_number, ind1 allele1, ind1 allele2, ind2 allele1, etc

- All individuals should be specified for all markers, unknown markers coded as 0
- chromosomes must have numbers rather than names and markers should be arranged by chromosome 1, 2, …

```
Example 1:
chr1
1  2 2 2 2
2  2 2 2 2
3  2 2 2 2
4  2 2 2 2
5  2 2 2 2
chr2
1 1 2 1 2
2 3 4 3 4
3 3 1 3 1
4 4 1 0 0
5 3 1 1 4
```

```
Example 2:
chr1
1  2 2 2 2
2  2 2 2 2
3  2 2 2 2
4  2 2 2 2
5  2 2 2 2
chrx
1  1 2 1 2
2  3 4 0 3
3  3 1 0 1
4  4 2 3 2
5  3 1 0 0
```

1 and 4 should be females if sscx is sex chr and first marker is in the PAR

```
Example 3: Transposed example
1 2 3 4 5
snp1 1 2 3 4 3 1 2 0 0 4 1
snp2 1 2 3 4 3 1 2 0 0 1 1
snp3 1 1 3 4 3 1 2 0 0 4 4
snp4 2 2 3 4 2 1 2 0 0 4 4
snp5 2 2 3 4 3 1 2 1 1 4 1
```

USER DEFINED COVARIANCE MATRIX FILES

Qxpak allows inclusion of random effects distributed as N(0,V) where V can be any positive definite matrix, and this matrix (only the upper diagonal) is read from a file. This matrix is inverted to obtain random effects predictions, then, the user can specify either that the file contains the inverse (saving computation) or the direct matrix. If the inverse is specified, the parameter file (EFFECT section) should look like

```
EFFECT
name_eff1 cross 1 g_usr file.inverse
name_eff2 cross 4 g_usr_inv file.direct
```

The effect 1 applies to the individual (column 1) and the file contains the inverse. The effect 2 applies to an effect in column 4 and contains the direct matrix. The format of the files is:

row, column, value

- Only nonzero elements need to be specified.
- Only upper diagonal (column ≥ row)
- The order of appearance of the elements is irrelevant.

HAPLOTYPE FILE

Contains known haplotypes if any. The format is

First record: name_of_chromosome
Successive records: individual, order of markers where phases known

- If several chromosomes are analyzed, this format should be repeated for each one.

Example:

```
chr1     !phases of marker 3 of ind 1 are exactly as read in marker file
1 3      !i.e., the first allele is of father's origin, the 2nd allele, mother's
3 2 3    !idem for ind 3, markers 2 & 3
chr2
4 1
```

OUTPUT FILES

The following files are written:

- *q.0*: contains running output that can be useful, e.g., to check convergence.

- output_file: contains main output, some examples are below. The name of the main output file is defined in the parameter file, otherwise the file is named 'qxpak.out' by default. Most of the results in the output file do not need special comment. However, P-values do merit special attention. P-values are nominal and are computed according the number of degrees of freedom estimated by the program. The program also provides P-values assuming other d.f. These are provided so that the user can choose more conservative (stricter) P-values.

- haplotypeoutput_file: if that section is specified, haplotypes sampled at each MCMC iteration are written. First records, for every individual, the list of markers with known (T) or unknown (F) phases. Known phases are not sampled. In sucessive records, the sampled phases are written for every iteration and individual. The format is:

  chromosome, MCMC_iteration, individual, phase, alleles

- 'z' files, like 'zran.10000', 'zmkr.2.1', 'zsnp.13', or 'zfix.380000'. They contain the IBD probabilities or SNP configurations.

- IBD files can be expensive to compute, so they are saved from run to run. They are recomputed only if the number of MCMC iterations or if the model is changed. You should not delete them and, specially, **you should NOT modify them**.
- For individuals with unknown parents, the phase order is arbitrary and the first heterozygous marker genotype is fixed for reference. Otherwise, first phase is male phase and second phase, female phase.

ADVANCED OPTIONS IN PARAMETER FILE

NUMBER_OF_MCMC_ITERATIONS (Optional, Default: 1000)
Used to compute IBD probabilities

SCAN_STEP (Optional, Default: 1)
In QTL scan, step in cM. Does not apply to association studies, where all SNPs are tested one at a time.

PRINT_SOLUTIONS (Optional, Default: Yes)
Specifies whether all solutions are printed (Yes) or only those corresponding to QTL effects (No). Sometimes, e.g., if you use the ran_ or mix_ options with many individuals, the solution output can be lengthy so this options reduces the amount of output and thus improves legibility. Yes, *Y, yes, or y are equivalent.* No, N, no, or n *are equivalent*

PRINT_RESIDUALS (Optional, Default: No)
If yes, the following four quantities are written for every record and trait:

$$y; \qquad \hat{e} = y - \mathbf{X}\hat{b} - \sum_{k=0}^{Nq} \mathbf{Z}\hat{\mathbf{g}}_k \qquad ; \qquad \sum_{k=0}^{Nq} \mathbf{Z}\hat{\mathbf{g}}_k \, ; \qquad y - \mathbf{X}\hat{b}$$

Residuals can be used for checking the adequacy of the model, or test for outliers; whereas $E(y-\mathbf{X}b) = \sum_{k=0}^{Nq} \mathbf{Z}\hat{\mathbf{g}}_k + \hat{e}$ can be used to have a rough idea of the QTL genotype for each individual, ie, it can be used approximately to distinguish homozygous individuals for each alternative allele. If the model is correct, this quantity should be similar to $\sum_{k=0}^{Nq} \mathbf{Z}\hat{\mathbf{g}}_k$.

HAPLOTYPEOUTFILE (Optional, Default: no haplotypes written)
Specifies the name of the file where sampled haplotypes are written. It allows checking whether phases are being sampled correctly, or to give probs. to any haplotype. The format of the haplotypeoutfile is given below.

Format
name_of_hap_file (first_haplotype, last_haplotype) (burnin #_burnin_iterates)

Default
- No haplotypes are written
- If no haplotype id is specified, all haplotypes are written
- If no burnin is specified, all MCMC iterates are written out

Example 1: all haps. for all iterates are written
hap_out

Example 2: haps. from all individuals after iterate 100 are written,
hap_out burnin 100

Example 3: haps. from inds. 12 trough 13 are written after iterate 300
hap_out 12 13 burnin 300

HAPLOTYPE_BLOCK_SIZE (Optional, Default: 6)
Integer, number of markers with phase updated simultaneously in the MCMC algorithm that computes IBD. Modify only if you have many markers and want to update in larger or smaller blocks, may cause numeric problems if too large. Use at own risk.

`COLLAPSE_ORIGINS (Optional, Yes/No, No default)`
Specifies whether all individuals within a breed are treated as clones (all IBD). This could be used when many breeds are specified. Yes means collapse origins is performed. **WARNING:** Option not tested. Email me if you want details.

`ACCURACY_POS section (Optional, Default constant accuracy)`
This section allows incorporating different reliabilities for each trait and individual. The residual variance for that observation is σ_e^2/Accuracy. This option is useful when phenotypes recorded are actually means but the number of observations used differ from record to record, this can be the case, e.g., in using means from recombinant inbred lines, and there are different individuals recorded in each line. This option can be useful also for dairy cattle, where a typical phenotype is daughter yield deviation and its accuracy differs from bull to bull. The respective column in the data file must contain the accuracy of the individual, which is the number of observations (1 for a single record). The format is:

trait_name, position_in_datafile_containing_accuracy_for_that_trait

This section must be after the TRAIT section, it is discarded otherwise. If MULTITRAIT option is true, same accuracy applies to all traits.

`INITIAL_RES_VAR (Optional, Default 0.6 phenotypic variance)`
Contains the initial residual variances in order variance_trait_1, cov_trait_1_2, ..,
variance_trait_2, cov_trait_2_1,

`INITIAL_GEN_VAR (Optional, Default 0.4 phenotypic variance)`
Format:
name of effect (first line)
initial values for full stored of variance/covariance matrix for that effect (second line)

This section must be repeated for each effect whose variance an initialization is required.

Example 1:
```
INITIAL_GEN_VAR
e1
4 5 5 16  <-- varTrait1, covTrait12, covTrait12, varTrait2
INITIAL_GEN_VAR
e2
2 3 3 7
```

Example 2:
```
INITIAL_GEN_VAR
e1
4 0 0 16  <-- Suppose that both traits are uncorrelated for effect e1
INITIAL_GEN_VAR
qtl2
2 0 3 0   <-- WRONG, unpredictable results
```

`INITIAL_QTL_VAR (Optional, Default 0.4 phenotypic variance)`
Same format and considerations as above. Meaningful only for ran_* and mix_* qtl modeling.

Example:
```
INITIAL_QTL_VAR
qtl_1
4 5 5 16  <-- varTrait1, covTrait12, covTrait12, varTrait2 for qtl_1
```

ADVANCED THEORY

Suppose two breeds, A and B, with genetic effects (g) normally distributed as $g_A \sim N(\mu_A, \sigma_A^2)$ and $g_B \sim N(\mu_B, \sigma_B^2)$, respectively. Now assume that a quantitative trait has been recorded in a population with an arbitrary pedigree complexity, where individuals can be 'purebred' from either A or B populations, F_1, F_2, or any other combination (e.g., recombinant inbred lines, backcross, advanced intercross, and so on). A general explicative model is

$$\mathbf{y} = \mathbf{Xb} + \sum_{k=0}^{Nq} \mathbf{Z}\,\mathbf{g}_k + \mathbf{e}, \qquad (1)$$

where **y** is a vector containing the recorded performances, **b** contains the fixed effects to be estimated, $\mathbf{g_k}$ contains the genetic (QTL) effects for any of the N_q QTL affecting the trait. By convention, we take $\mathbf{g}_0$ to stand for the infinitesimal genetic effects, i.e., the genetic effects not accounted for by individual QTL. Finally, **X** and **Z** are incidence matrices that relate observations to the parameters in the **b** and **g** vectors, and **e** is the residuals' vector.

The model in (1) is termed 'mixed' because it contains fixed effects, such as sex or age, and random effects, such as the genetic effects, **g**. Statistical theory for mixed models is well developed (McCulloch & Searle 2000) and theory dictates that we also have to specify the distribution of the random variables, i.e., their means and variances. In the case of the QTL effects, $\mathbf{g}_k$, the expected value of the i-th indivual at k-th locus is

$$E(g_{ik}) = P(g_{ik}^1 \in A, g_{ik}^2 \in A)\ \mu_{AAk} + P(g_{ik}^1 \in B, g_{ik}^2 \in B)\ \mu_{BBk} + [P(g_{ik}^1 \in A, g_{ik}^2 \in B) + P(g_{ik}^1 \in B, g_{ik}^2 \in A)]\ \mu_{ABk}. \qquad (2)$$

Here $P(g_{ik}^1 \in U, g_{ik}^2 \in W)$ is the probability that alleles from k-th QTL at paternal and maternal haplotypes are of breed U and W origins, μ_{WZk} is the mean genetic effect of individuals having received a U and W origin alleles at locus k. The variance of $\mathbf{g}_k$ is a matrix, $\mathbf{G}_k$, that contains the covariance between the i-th and j-th genetic values at k-th locus. The covariance between i-th and j-th genetic values is

$$Cov(g_{ik}, g_{jk}) = \frac{1}{2}\sum_{h=1}^{2}\sum_{h'=1}^{2} P(g_{ik}^h \equiv g_{ik}^{h'} \mid g_{ik}^h \in A)\,\sigma_{Ak}^2 + \frac{1}{2}\sum_{h=1}^{2}\sum_{h'=1}^{2} P(g_{ik}^h \equiv g_{ki}^{h'} \mid g_{ik}^h \in B)\,\sigma_{Bk}^2, \qquad (3)$$

where $P(g_{ik}^h \equiv g_{ik}^{h'} \mid g_{ik}^h \in U)$ is the probability of alleles g_{ik}^h and $g_{jk}^{h'}$ being identical by descent (IBD) and from origin U, superscript h stands for the paternal or maternal phases, numbered 1 or 2, respectively, and σ_{Uk}^2 is the variance of genetic effects of U origin at locus k.

Multiple breed analyses: Qxpak allows for any number of breed origins. That is,

$$E(g_{ik}) = \sum_U P(g_{ik}^1 \in U)\,\mu_{Uk} + \sum_U P(g_{ik}^2 \in U)\,\mu_{Uk}$$

and

$$Cov(g_{ik}, g_{jk}) = \frac{1}{2}\sum_U \sum_{h=1}^{2} \sum_{h'=1}^{2} P(g_{ik}^h \equiv g_{ik}^{h'} \mid g_{ik}^h \in U)\,\sigma_{Uk}^2$$

Here no dominance is allowed, ie, the options fix_ad, fix_d (see below) and the like are not allowed for. The program automatically identifies how many breeds are defined from the pedigree file. By default, one breed is assumed.

Sex linked QTL: A particular case occurs when the QTL lies in the differential part of the sex chromosome (X in mammals, Z in birds). At least in mammals, but also probably in birds (Ellegren 2002), dosage compensation exists, which means that genetic effects are different according to whether the QTL allele lies in a female or in a male. We have proposed to include a dosage compensation parameter, ψ, to account for this differential effect. According to this model, the expected genetic value of males is:

$$E(g_{ik}) = P(g_{ik}^2 \in A)\ \mu_{AAk} + P(g_{ik}^2 \in B)\ \mu_{BBk},$$

note that males have only one allele and is that received from the mother. In the case of females,

$$E(g_{ik}) = \psi\ [\ P(g_{ik}^1 \in A, g_{ik}^2 \in A)\ \mu_{AAk} + P(g_{ik}^1 \in B, g_{ik}^2 \in B)\ \mu_{BBk}\ ] + [P(g_{ik}^1 \in A, g_{ik}^2 \in B) + P(g_{ik}^1 \in B, g_{ik}^2 \in A)\ ]\ \mu_{ABk},$$

i.e., the genetic effect of an allele in a female is weighed by ψ, with respect to the effect of the same allele it it happens to be in a male. Logically, interaction between alleles (dominance) can be estimated only in females. We also need to define different genetic covariances according to sex. It can be shown that the genetic covariances between any two crossed individuals are:

$$Cov(g_{ik}, g_{jk}) = Pr\,(g_i^2 \equiv g_j^2 \in A)\,\sigma_{Ak}^2 + Pr\,(g_i^2 \equiv g_j^2 \in B)\,\sigma_{Bk}^2$$

if i and j are males;

$$Cov(g_i, g_{i'}) = \sum_{h=1}^{2} \psi^h\ [\ Pr\,(g_i^2 \equiv g_j^h \in A)\,\sigma_{Ak}^2 + Pr\,(g_i^2 \equiv g_j^h \in B)\,\sigma_{Bk}^2\ ]$$

when i is a male and j is a female; and

$$Cov(g_i, g_{i'}) = \sum_{h=1}^{2} \sum_{h'=1}^{2} \psi^h\ \psi^{h'}\ [Pr\,(g_i^h \equiv g_j^{h'} \in A)\,\sigma_{Ak}^2 + Pr\,(g_i^h \equiv g_j^{h'} \in B)\,\sigma_{Bk}^2\ ],$$

for both i and j being females. QxPak accommodates sex chromosomes setting $\psi = 0.5$. This parameter cannot be modified (contact the authors if you wished this parameter changed).

Imprinting is a somewhat related issue to sex linked traits from a modeling point of view. We discourage the use of the imprinting options in QTL analysis because of the difficulty in interpreting the results. Imprinting means that either the paternal or maternal alleles are expressed, the other allele being inactivated by metilation. Imprinting pattern can be very diverse, it can change through development, according to tissue, and so on. Thus, with respect to a given phenotype, it is risky to infer what are we recording. Furthermore, the only way of really checking whether there is imprinting is to compare heterozygous individuals for the causal mutations where the origin of the allele can be unambigously determined. If, despite these warnings, you are willing to use the imprinting option, you should know how Qxpak allows for imprinting. This is done following modelization

$$\mathbf{y} = \mathbf{Xb} + \mathbf{Zu} + \mathbf{g}^1 + \mathbf{e} \qquad (4a)$$

for maternal imprinting (i.e., only the allele of paternal origin is expressed) or

$$\mathbf{y} = \mathbf{Xb} + \mathbf{Zu} + \mathbf{g}^2 + \mathbf{e} \qquad (4b)$$

for paternal imprinting. Qxpak allows imprinting for any QTL modeling, i.e., fix_, ran_, snp_ or ld_ are allowed for. Two aspects should be noted. First, models (4) and (1) are NOT nested and thus our modeling does not allow for likelihood ratio tests to test for imprinting. Nevertheless, a large difference in likelihood in favor of model (4) could be taken as suggestion of imprinting, with all cautions above. Second, Qxpak considers only records for which the origin of the allele can be determined unambigously given parentals' genotypes.

A different issue to sex linked QTL is sex x QTL interaction which, incidentally, has been shown to be a common phenomenon, specially for fitness related traits. QxPak also allows for testing this interaction or, in general, any interaction between a covariate and a class effect. For instance, QTL x environment interaction, a quite popular topic of discussion in plant breeding, can be tested with QxPak if the environment can be classified in classes, e.g., seasons, localization, herd-year-season, etc.

Likelihood computation: In order to compute the likelihood, we still need to define additional distributions. The distribution of all random variables in model (1) is:

$$\begin{pmatrix} \mathbf{y} \\ \mathbf{g} \\ \mathbf{e} \end{pmatrix} \sim \mathrm{N}\left[\begin{pmatrix} \mathbf{Xb} + \mathbf{P\mu} \\ \mathbf{P\mu} \\ \mathbf{0} \end{pmatrix}, \begin{pmatrix} \mathbf{V} & \mathbf{GZ'} & \mathbf{R} \\ \mathbf{ZG} & \mathbf{G} & \mathbf{0} \\ \mathbf{R} & \mathbf{0} & \mathbf{R} \end{pmatrix} \right],$$

where **P** is a matrix containing the $\mathrm{P}(g_{ik}^1 \in U, g_{ik}^2 \in W)$ elements described in equation (2), μ is a vector with the μ_{Wk} elements for every QTL, **V** = Var(**y**) = **ZGZ'** + **R**. The matrix **R** contains the variances and covariances of the residuals. This matrix is diagonal in univariate models and block-diagonal for multitrait analyses. In the case of missing traits, these blocks are different for each individual according to which trait(s) is(are) missing. Matrix **G** is equal to $\sum_{k=0}^{Nq} \mathbf{G}_k$, each matrix $\mathbf{G}_k$ is a matrix made up of the terms described in eq. (3). Here it is assumed that the total genetic variance is the sum of the variances at each locus. This is an approximation when there is linkage disequilibrium in the outbred populations, but is accurate as long as markers are not very sparsely located.

In order to compute the likelihood and carry out standard statistical tests, it suffices to obtain quantities (2) and (3) at any desired genome positions for all individuals and plug them into the likelihood function. It is important to notice that exactly the same computing strategy is followed irrespective of the pedigree complexity, number of QTL or traits. For instance, a cross between inbred lines can be modelled setting all elements in (3) to zero. Alternatively, we set elements in (2) to zero in a within population analysis because all genetic values have the same mean, logically, we need to estimate a single genetic variance $\sigma^2 = \sigma_A^2 = \sigma_B^2$ in (3). If we are interested in testing imprinting, the same formulas hold but the maternal (h=2) or paternal (h=1) coefficients P(.) are set to zero.

QTL modeling in Qxpak follows the theory explained above. The simplest option, when analyzing a cross between two inbred lines, is to define a QTL, say qtl1, as **fix_a** or **fix_ad**.

- **fix_a**: means that $\sigma_A^2 = \sigma_B^2 = 0$, and the only parameter being estimated is $\mu_{kAA} - \mu_{kBB}$, i.e., the genetic difference between breed A and breed B alleles; μ_{kAB} is also set to zero.

- **fix_ad**: is as fix_a, except that μ_{kAB}, the dominance effect, is estimated. Dominance effect can be tested via a likelihood ratio test between the two models. The program allows to do this automatically in the TEST section (see below). You could write

  TEST
  qtl1 fix_a

Similarly, you can fit only the dominance effect using the **fix_d** option, although this option is included more for completeness than for its biological significance. Now suppose you an outbred population is being analyzed. Then you are most likely interested in estimating the amount of additive genetic variability explained by a genome region. You can model the QTL as **ran_1**, where subscript '1' means that you are setting $\sigma_k^2 = \sigma_{Ak}^2 = \sigma_{Bk}^2$ in eqn. (3).

- **ran_1**: means that you set the expectation of all QTL alleles to 0. Implicitly, you are assuming that there are as many alleles as twice the number of founders in the pedigree (much as in any infinitesimal model heritability analysis) and that you are not interested in each allele effect individually but rather in the global contribution of that region to the whole phenotypic variability. Allelic effects are treated as random. You can test whether σ_k^2 is zero typing in the parameter file:

  TEST
  qtl1

  The program then computes the likelihood deleting qtl1 from the model. An important detail here is that the number of degrees of freedom is here halved with respect to the previous case because σ^2 can only take positive values. What does this mean? Suppose that the LRT is 3.84 and has 1 d.f., which corresponds to a P-value of 5% in a typical setting, if you are testing a random effect, the approximate P-value is 2.5% instead.

Now consider the most complex situation. Suppose that you have a cross of two populations but it cannot be ensured that they are completely inbred. Similarly, you can not be sure that the heritability in both breeds is the same. You can model qtl1 as:

- **mix_2a**: means that the values of QTL effects are, on average, different between breeds and that there is variability within breeds. This variability can be different between breeds. In the simplest situation, we assume that there is no dominance.

- **mix_1a**: we make the further simplification that the genetic variability is the same for both breeds at that particular locus.

- **ran_2**: means that expected values are the same ($\mu_{kAA} = \mu_{kBB} = \mu_{kAB} = 0$) in both breeds but still the genetic variance within both breeds is different.

It is also possible that there exists genetic variability within one breed but not the other. This can be accommodated via the _01 and _10 options:

- **ran_01**: means that there is no variability in breed 1 for that QTL (σ_{Ak}^2 = 0).
- **ran_10**: means that there is no variability in breed 2 for that QTL (σ_{Bk}^2 = 0).
- **mix_01a**: same as **mix_2a** with σ_{Ak}^2 = 0.
- **mix_10a**: same as **mix_2a** with σ_{Bk}^2 = 0.
- **mix_01d**: same as **mix_2d** with σ_{Ak}^2 = 0.
- **mix_10d**: same as **mix_2d** with σ_{Bk}^2 = 0.
- **mix_01ad**: same as **mix_2ad** with σ_{Ak}^2 = 0.

- **mix_10ad**: same as **mix_2ad** with σ^2_{Bk} = 0.

The options can be useful because sometimes crosses are carried between highly divergent breeds, and may be one is highly inbred but not the other. Using the **_2** options in this circumstance may lead to numeric problems as one variance is estimated to zero. Similarly, the **_1** options are not realistic. Likelihood ratio tests can be done between the **_2** and **_01** or **_10** options.

Modeling dominance in crosses between outbred populations is extremely difficult in a classical paradigm. We have implemented option **mix_2ad** and **mix_1ad** to accommodate, approximately, dominance, but the user should be aware that we are this is an approximation and not a fully correct method from a theoretical point of view. If you find strong evidence of variability within breeds and results change dramatically between a mix_1ad and mix_1a models (or mix_2ad and mix_2a), be aware that results can be misleading.

All QTL types described above can be fitted using either a genetic scan between predelimited bounds or fitting a **segment** that comprises all the genome between these bounds. In a QTL scan, the model is fitted as successive intervals say every cM and the most plausible location is identified as that of maximum likelihood. This is the classical strategy. The segment approach consists of partitioning the genome in a series of segments, delimiting specific genome regions that we are interested in analyzing. The method allows us to model the segment as a fixed effect, like in crosses between inbred lines, as a random effect, eg in purebred analysis, or as a mixed model, ie, the same options as in the QTL scan are allowed. The fraction of total genetic variance explained by each segment is subsequently estimated. In this approach, the most relevant question is the fraction of genetic variance explained by the whole segment, we are not interested so much in how many quantitative trait loci (QTLs) are there or in their exact position. To specify a QTL as segment, the term segment (or seg for short) must be written after the QTL type definition and before the QTL positions. By default, a QTL is scanned.

Association studies: One of the most active areas of research in QTL studies is the discovery of causal mutations through the scan of multiple SNPs or genome wide association studies (GWAS). Here the issue of interest is assessing whether a polymorphism has an effect *per se* on the trait, i.e., if there exists an association (linkage disequilibrium) between the SNP genotype and the trait. QxPak allows the automatization of multiple SNP tests. Similar to **fix_*** options, you have three **snp_** possibilities:

- **snp_a**: individuals with marker genotypes '11', '12' and '22' are assumed to have genetic values μ_{kAA} , 0 and μ_{kBB}, respectively. Note: genotypes '12' and '21' are treated identically.

- **snp_ad**: individuals with marker genotypes '11', '12' and '22' are assumed to have genetic values μ_{kAA} , μ_{kAB} and μ_{kBB}, respectively.

- **snp_d**: individuals with marker genotype '11', '12' and '22' are assumed to have genetic values 0, μ_{kAB}, and 0, respectively.

Note that, in the SNP modeling, we are estimating the effect of the allele itself whereas the marker alleles are used solely to identify the breed origin of a crossed individual in the fix_ options. Thus, contrary to the fix_ options, you do not need to specify two breeds with SNPs. QxPak computes the likelihood with every SNP in turn, and that with the maximum likelihood is retained. Note: allele codes must be '1' and '2', the program crashes if there are more than two alleles. An allele code '0' means the genotype is missing.

We have implemented two additional ways of analyzing association between markers and phenotypes suitable for multiallelic markers:

- **ld_fix**: an additive effect is estimated for each allele. Suppose individuals have genotypes a_1a_2, a_1a_3 and a_3a_3, the model would be:

$$y_1 = Xb + a_1 + a_2 + e$$
$$y_2 = Xb + a_1 + a_3 + e$$
$$y_3 = Xb + 2a_3 + e$$

- **ld_ran**: same as ld_fix except that allelic effects are treated as random, i.e.,

$$\begin{pmatrix} a_1 \\ a_2 \\ a_3 \end{pmatrix} \sim N\left[\begin{pmatrix} 0 \\ 0 \\ 0 \end{pmatrix}, \begin{pmatrix} 1 & 0 & 0 \\ 0 & 1 & 0 \\ 0 & 0 & 1 \end{pmatrix}\sigma^2_\alpha\right]$$

Neither ld_fix nor ld_ran allow for dominance. **Note**: snp_a and ld_fix with biallelic markers is exactly the same parameterization.

Molecular relationship matrix and association in structured populations: A recognized major risk in association studies are false positives caused by structure, i.e., heterogeneity within the sample. A first attempt to decrease this risk was to use principal components, but recently mixed models have been shown to be more powerful and reliable. In this setting, a random genetic effect **g** ~ N(0, **G**) can be included in the model together with the SNP direct effect. Above, **G** is an IBD matrix that can be obtained in a number of algorithms. In Qxpak, two options are implemented. The first one is

G =**MM'**/n,

where **M** is a m individuals x n SNPs matrix with values -1, 0 and 1 for genotypes 11, 12 and 22, respectively. Note that **MM'** contains the scaled number of alleles shared between individuals, averaged over markers. The program automatically considers heterogametic sex markers. In this case, males are considered to be equivalent to homozygous females. The standardized matrix **G** can also be computed setting option `STANDARDIZED_MOL` to `YES` in the parameter file:

$\mathbf{G^*} = \mathbf{WD^{-1}W'}/n$,

where $\mathbf{W} = \{w_{ij} = (m_{ij} - \mu_j)\}$ where μ_i is the genotypic mean frequency of i-th SNP ($2q_i$ -1), q_i being the frequency of allele 2, and **D** is a diagonal matrix with elements $2q_i(1-q_i)$, the genotype variance. This is the option considered, e.g., in Yang et al. (2010) except that they employ a slight variant for the diagonal elements. The user can also accommodate any desired positive definite matrix using the `g_usr` options in the `TRAIT` section. Either the direct or the inverse matrix can be employed (computationally, it is much more efficient to input the inverse).

Since the first version of Qxpak, we advocated for using an infinitesimal genetic effect in the model. In fact, if the pedigree were known for all individuals included in an association study, the infinitesimal genetic effect would correct as well for structuring. Qxpak allows to define the marker IBD matrix using any given set of markers, all markers genome wide or markers chromosome by chromosome.

Modeling epistasis can be done between loci that are defined as fix_ (in crosses between pairs of breeds), snp_ (association study), or ran_ (epistatic covariance matrix). Suppose we have the usual table for two loci, A and B

	Locus 2		
Locus 1	BB	Bb	bb
AA	P_1	P_2	P_3
Aa	P_4	P_5	P_6
aa	P_7	P_8	P_9

In a cross between inbred lines (option epi_fix), the program computes the joint probabilities, P_i. For instance P_4 is the probability that the individual is heterozygous for alleles from the two breeds at the first locus and homozygous for breed B origin in locus 2. In a usual Cockerham's decomposition the four components are estimated by regressing on a linear combination of these 9 probabilities.

additive x additive: $P_1 - P_3 - P_7 + P_9$
additive x dominant: $P_2 - P_8$
dominant x additive: $P_4 - P_6$
dominant x dominant: P_5

logically, the order dominant x additve vs. additive x dominant is arbitrary and depends on what you called locus 1 and locus 2. in Qxpak, the user can arbitrarily modify these coefficients to accommodate any linear combination of the P's using the custom keyword (see below).

The next option (epi_snp) is when you have two pairs of SNPs and you want to test for interaction between. The same table appears but notice that here we test directly the haplotype effect. In the table below, $\lambda_i = 1$ means that the individual has i-th haplotype.

	Locus 2		
Locus 1	BB	Bb	bb
AA	λ_1	λ_2	λ_3
Aa	λ_4	λ_5	λ_6
aa	λ_7	λ_8	λ_9

By default Qxpak pools genotypes (1, 3, 7, 9), (2, 8), (4, 6) and (5) and estimates an effect for this pooled haplotypes, here 4 levels. However, the user can modify this using the custom keyword (see below). For instance, one could test whether genotype *aabb* is significantly different from the 8 others.

Finally, option epi_ran allows to test for epistasis between two or more random QTL. This is the only option that accommodates epistasis between more than two loci. For this option, a new IBD QTL covariance matrix is computed whose elements g_{ij}^{epi} are simply the product of the elements of the individual IBD matrices, i.e, $g_{ij}^{epi} = \prod_{k=1}^{nloci} g_{ij}^{k}$. Note that this way of modeling epistasis, proposed by Blangero et al, can be a bit controversial. For instance, if there is inbreeding the diagonal elements of the IBD matrix are > 1, and the joint probability increases rather than decreasing. I need to think about this a bit more.

Caution: QxPak implements as many QTL (or markers via snp_ or ld_) as desired and each can be modelled independently, allowing far greater flexibility than most currently available

programs. However, there is a price to pay for adding new QTL, as the number of positions to be evaluated increases geometrically with number of QTL.

An additional important option of QxPak is that multitrait options are fully implemented. Multivariate mixed model techniques are well known and developed but have not been applied yet to QTL analyses, although least squares approaches have been published. The advantage of mixed model methods, again, lies in its flexibility compared to least squares. For instance, mixed model theory can accommodate easily missing data, as well as different models per trait, which is not possible in a least squares' approach. In a multivariate setting, we need to define the QTL variances for each trait plus their covariances, that is $\mathbf{G}_{trait1,trait2} = \mathbf{G}_0 \otimes \begin{pmatrix} \sigma_{11}^2 & \sigma_{12} \\ \sigma_{12} & \sigma_{22}^2 \end{pmatrix}$, where $\mathbf{G}_0$ contains the P(.) coefficients in eqn. (3), ⊗ stands for the Kronecker product, the σ_{ij}^2 term is the genetic covariance between traits i and j. We have assumed for the sake of simplicity a within breed analysis ($\sigma_{ij}^2 = \sigma_{Aij}^2 = \sigma_{Bij}^2$) but the same principle applies for separate breeds.

One of the interests of fitting multitrait models lies in disentangling whether a QTL that affects two traits simultaneously is due to a single QTL (pleiotropy hypothesis) or to two distinct QTL (linkage hypothesis) that lies in the same genome region. When the QTL effects are modelled as fixed, (σ_k^2 = 0), these two alternatives can be tested by fitting the same QTL for both traits or fitting two distinct QTL, one for each trait. The difference in likelihoods between the two competing models can be tested using a likelihood ratio test with one degree of freedom if only the additive effect is fitted or two if also the dominant effect is included. This can be done easily with our software. The issue of the significance level is discussed below. It should be noted, however, that the situation is more complex when the QTL effects are random: the linkage and pleiotropy models are not hierarchical in this case and thus the likelihood ratio test cannot be applied. This occurs because the pleiotropy model contains the locus position, the genetic variances plus the covariance between traits, whereas the linkage model contains two loci positions but does not include the genetic covariance. Thus, other criteria must be used like the Bayesian information criterion or Akaike's information criterion.

Unprecedented amount of data provided by microarray technology has been accompanied by numerous studies to dissect the genetic basis of each of the mRNA levels measured. One of the requirements, thus, will be to automate numerous QTL studies, and QxPak allows for this if the MULTITRAIT option is on. As with any other quantitative trait, the same modeling flexibility applies, e.g., an infinitesimal genetic effect can be fitted, or several QTL if desired. The only limitation is that the same model should be used for all traits (mRNA levels). This is due to input simplicity rather than computing constraints because it is difficult for the user to specify thousands of potentially different models.

Hypotheses tests

Hypothesis testing has always been a controversial issue in QTL analyses. Permutation tests have become quite popular because they are robust, they do not require any distribution assumptions, and are simple to apply. The idea behind permutation tests is to shuffle phenotypes with respect to marker information in order to mimic the null hypothesis of no association between marker and phenotype. Although Qxpak initially implemented permutation, we have abandoned that for reasons detailed below and increasing complexity of the program. and this option is not fully tested (it could be implemented via a perl script though). As a rule of thumb, we recommend a P-value of 0.001.

Permutation tests have been implemented via the PERMUTATIONFILE section in the parameter file. However, this is no longer supported and may not apply to all modeling options. It is VERY important to know how permutation is implemented in Qxpak: we shuffle IBD probabilities with respect to individuals and phenotypes. In the simplest setting, this is

equivalent to the usual phenotype shuffling, however, permutation tests can not be used to test other effects rather than the QTL effects (e.g., cannot be used for testing sex, batch, etc). Moreover, permutation tests can be used only to test all QTL simultaneously, that is, we cannot test a two QTL vs a one QTL model. The interpretation of permutation with infinitesimal genetic effects is also problematic.

Thus, despite its deceiving simplicity, permutation tests cannot be easily applied to any situation. For instance, suppose an infinitesimal genetic effect has been included in the null model and one is interested in testing the importance of adding a QTL effect. If data are permuted, the pedigree structure is broken making the estimate of the infinitesimal genetic effect meaningless. Thus, with permutation, one is testing jointly the effects of the QTL and the infinitesimal effect. Similar problems arise when random effects should be tested, in multitrait models, or simply in two QTL versus 1 QTL tests.

Traditionally, likelihood ratio tests (LRT) have been used in maximum likelihood inference. It consists of computing minus twice the difference in log-likelihoods between the alternative and the null models. Asymptotically, this quantity is distributed as a Chi-square with degrees of freedom equal to the difference in the number of parameters between the alternative and the null models. QxPak provides the likelihood ratios under the models tested and the associated nominal P-values. The program automatically detects the difference id degrees of freedom, but the user should check that the estimated difference in d.f. is correct, as not all possibilities have been fully tested.

It has long been recognized that a much more strict significance threshold should be used to account for the genome scan process. This means that a P-value of 5% as obtained from the chi-square approximation will not normally be accepted as significant in a QTL scan. Thus, we recommend the chi-square approximation for the likelihood ratio test but with a more strict P-value than the nominal significance level, along the guidelines published. All in all, P-values bordering significance should be looked up cautiously, as there may be many approximations involved. The program provides nominal P-values with different degrees of freedom. The first P-value corresponds to the least conservative approach, however, at least one d.f. should be added for every QTL in the model to allow for the extra parameter of each position.

Nevertheless, setting the 'correct' significance threshold is a difficult issue. In a former study we showed that the Chi-squared with 2 d.f. corresponding to the 5% and 1% chromosome wise significance P-values were 10.5 and 14.2, these figures in turn correspond to nominal P-values of 0.005 and 0.001, roughly. Although we cannot claim that this is a completely satisfactory solution, we can reasonably argue that nominal P-values smaller than 0.001 are significant for the single QTL test. The user can always use permutation, with the caveats outlined above.

A final, perhaps obvious, remark: it must be remembered that LRT are applicable only to hierarchical tests, that is, the null model must contain all parameters of the alternative model. For instance, if the alternative model contains effects QTL and sex, the null model cannot be one with sex and age as effects.

One vs. two or more QTL

Fitting two or more QTL is straightforward in Qxpak. However, these options should be used cautiously. We recommend that a single QTL per chromosome to be fitted initially. Sometimes a double peak in the P-profile can appear, suggesting that two nearby QTL can be segregating. However, a double mode can be the result of a not too informative marker in between. Generally, fitting two QTL in these instances may result in weird estimates, like one qtl in position 30 (say) and the other one in the next position (31) with opposite effects. It is unlikely that two QTL in the same marker interval can be distinguished from a model with a single QTL. These statements will result in computing the LRT between a one QTL vs a two QTL model.

```
QTL
qtl_1 fix_a chr1 0 40
qtl_2 fix_a chr1 41 120
TRAIT
trait_name pos_trait qtl_1 qtl_2
TEST
qtl_2
```

The issue of which is the reasonable significance P-value has been much less studied than with a single QTL tests, but the same concerns apply. That is, unless we have biological evidence to the contrary, we should be conservative.

Testing pleiotropy vs. linkage

Suppose you want to test one qtl affecting two traits or a two qtl (pleiotropy vs linkage), you can specify

```
QTL
qtl_1 fix_a global
qtl_2 fix_a global

TRAIT               ! in run 1 (pleiotropy)
trait_1 pos_trait_1 qtl_1
trait_2 pos_trait_2 qtl_1

TRAIT               ! in run 2 (linkage)
trait_1 pos_trait_1 qtl_1
trait_2 pos_trait_2 qtl_2
```

Algorithm and implementation

The algorithm consists of two main steps: first, the IBD probabilities, i.e., the terms P(.), in eqns. (2) and (3), are computed and second, the likelihood is maximized. The algorithm used to compute IBD probabilities is a Monte Carlo Markov Chain algorithm largely based on the ideas presented (Pérez-Enciso *et al.* 2000) but with significant improvements: the algorithm updates several marker phases simultaneously thus making convergence faster, specially when markers are tightly linked; and missing marker information is allowed for by using the closest informative marker available. This applies only to linkage analyses options and not to association studies.

The IBD files are saved to disk so they can be reused in later analyses, saving computing time. These files are named with names starting by 'z', e.g., zfix.10000, zran.1300000, zsnp.133, zsni.22 or zrai.1. The marker relationship matrices are stored in files called zmkr. The algorithm consists of the following steps:

1. For each marker and individual, sample phases, conditional on the other current phases and markers. If a given marker is uninformative or missing, the next informative one is taken.

2. Sample crossovers conditional on phases and genotypes.

3. Track genome origins from founders and compute IBD states.

IBD coefficients depend on the genome positions at which they are fitted. The algorithm first identifies all possible positions needed and compute a likelihood for every combination, e.g., if there are three QTL fitted, each in one chromosome of lengths 40, 80 and 60 cM and the positions are scanned every cM, there will be 40 x 80 x 100 = 240,000 different likelihoods to be maximized.

In the second step, the mixed model equations are built and maximum likelihood estimates are obtained via the EM-algorithm. The mixed model equations are:

$$\begin{pmatrix} \hat{\mathbf{b}} \\ \hat{\mathbf{g}}_0 \\ \ldots \\ \hat{\mathbf{g}}_{Nq} \end{pmatrix} = \begin{pmatrix} \mathbf{X'R^{-1}X} & \mathbf{X'R^{-1}Z} & .. & \mathbf{X'R^{-1}Z} \\ \mathbf{Z'R^{-1}X} & \mathbf{Z'R^{-1}Z} + \mathbf{G_0^{-1}} & .. & \mathbf{Z'R^{-1}Z} \\ .. & .. & .. & .. \\ \mathbf{Z'R^{-1}X} & \mathbf{Z'R^{-1}Z} & .. & \mathbf{Z'R^{-1}Z} + \mathbf{G}_{Nq}^{-1} \end{pmatrix}^{-1} \begin{pmatrix} \mathbf{X'R^{-1}y} \\ \mathbf{Z'R^{-1}y} \\ \ldots \\ \mathbf{Z'R^{-1}y} \end{pmatrix}$$

(Henderson 1984,McCulloch & Searle 2000), where $\hat{\mathbf{b}}$ contains the best linear unbiased estimates of the fixed effects, whereas $\hat{\mathbf{g}}_k$ contains the best linear unbiased predictors of the k-th QTL genetic effects. These quantities are required to compute the QTL and residual variances, eqns. (4) and (5) below. Typically, $\mathbf{Z'R^{-1}Z}$ is a diagonal matrix with elements 1 at position (i,i) if i-th individual has a record, 0 otherwise. If there are several traits or repeated measures for the same individual and trait, $\mathbf{Z'R^{-1}Z}$ is block diagonal.The EM formulas for single traits are:

$$\hat{\sigma}_k^2 = \frac{\hat{\mathbf{g}}_k{}'\mathbf{G}_k^{-1}\hat{\mathbf{g}}_k + \hat{\sigma}_e^2 \,\mathrm{tr}[\mathbf{G}_k^{-1}(\mathbf{Z'Z} + \mathbf{G}_k^{-1}/\hat{\sigma}_k^2)^{-1}]}{n_k}, \qquad (4)$$

where $\hat{\sigma}_k^2$ is the estimate of genetic variance for the k-th random effect, $\hat{\mathbf{g}}_k$ contains the prediction of the k-th QTL genetic values (see Appendix), $\hat{\sigma}_e^2$ is the residual variance estimate, 'tr' stands for trace (Searle 1982), and n_k is the rank of $\mathbf{G}_k$ matrix (i.e., the number of individuals in the pedigree). The residual variance is estimated via

$$\hat{\sigma}_e^2 = \frac{\mathbf{y}'(\mathbf{y} - \mathbf{X}\hat{\mathbf{b}} - \sum_k \mathbf{Z}\,\hat{\mathbf{g}}_k)}{n_r}, \qquad (5)$$

where $\hat{\mathbf{b}}$ contains the estimates of the fixed effects (see Appendix) and n_r is the number of records. These two formulas need to be applied iteratively until convergence. These formulas are easily extended to multivariate models when the individual has measures for all traits. When there are missing traits, the formulas are more complex, see documentation of BLUPF90 package described below. QxPak fully supports multivariate models with any pattern of missing data, as it has inherited these properties from REMLF90 (Misztal et al. 2002).

The program also provides the likelihood, which can be used to test the desired effects (e.g., 2 QTL vs 1, or one QTL vs. none) via a likelihood ratio test. Minus twice the log-likelihood is given by

$$-2\ln(L) = \text{constant} + \ln|\mathbf{R}| + \ln|\mathbf{G}| + \ln|\mathbf{C^R}| + \mathbf{y}'\,\mathbf{R}^{-1}(\mathbf{y} - \mathbf{X}\hat{\mathbf{b}} - \sum_k \mathbf{Z}\,\hat{\mathbf{g}}_k)$$

where **R** and **G** are the variance of the residuals and of the genetic effects, respectively, $\mathbf{C^R}$ is the submatrix of the MME coefficient matrix that corresponds to the random effects, inverted:

$$\mathbf{C^R} = \begin{pmatrix} \mathbf{Z'R^{-1}Z} + \mathbf{G_0^{-1}} & \ldots & \mathbf{Z'R^{-1}Z} \\ \ldots & \ldots & \ldots \\ \mathbf{Z'R^{-1}Z} & \ldots & \mathbf{Z'R^{-1}Z} + \mathbf{G}_{Nq}^{-1} \end{pmatrix}^{-1}.$$

Programming is in Fortran 95 and we use modules from package BLUPF90 (Misztal *et al.* 2002), available at http://nce.ads.uga.edu/~ignacy/newprograms.html. These modules were designed to simplify operations with sparse and dense matrices. Module SPARSEM supports selected operations on sparse matrices, creation of a matrix, extracting blocks, solving, and computing traces and quadratic forms. Module FSPAK90, which is an interface to FSPAK written in Fortran 77 (Pérez-Enciso *et al.* 1994), supports sparse Cholesky decomposition, sparse finite solving and sparse inversion. In the implementation, the left hand side of the MME and each $\mathbf{G}_i^{-1}$ were stored as sparse matrices. Solutions to the mixed model equations were obtained via sparse Cholesky factorization. Calculation of the trace involved the use of a sparse inverse as detailed (Misztal & Perez-Enciso 1993).

ACKNOWLEDGMENTS

Work funded by the Ministry of Research and Education in Spain through several grants since 2004. Thanks to A. Legarra, J.P. Gutiérrez, I. Aguilar, B. Yang, Y. Ramayo, A. Reverter, M.A. Toro, H.B. Park, J. Estellé for subroutines, debugging, testing the program or comments.

APPENDIX 1: EXAMPLES

There should be a list of example files in the directory.

EXAMPLE 1 (qxpak_1.par): Simple F2 cross between inbred lines

This example illustrates a typical QTL linkage analysis. We have defined a single qtl as fixed, and thus, we need to have two breeds that are specified in the qxpak_1.ped file (5th column). The TEST section specifies that the null model is a model containing only the mean , infinitesimal effect and age, and the alternative model contains, in addition, the QTL. The solution vector is arranged by order of effect as defined, first are qtl. The first solution is the additive effect (breed 1 minus breed 2), the dominance effect, age covariate and infinitesimal effect estimates (there are 11 individuals in pedigree).

EXAMPLE 2 (qxpak_2*.par): Simple within population analysis

This example illustrates the dangers of not fitting an infinitesimal model in analyzing outbred populations. The first analysis simply estimates h2. The first parameter file tests the qtl effect in a model that already includes the infinitesimal genetic value. We suggest that this should be the null hypothesis. If a random QTL is fitted without the infinitesimal genetic value, the QTL is significant because it captures the whole genetic variance.

EXAMPLE 3 (qxpak_3.par): Simple multitrait batch

Here we use the same dataset as in example 1 except that we run three traits automatically, those in positions 5 through 8. Note that you need to specify the number of traits right after declaring 'yes' in the MULTITRAIT_OPTION, otherwise the program crashes. The NUMBER_OF_TRAITS section is irrelevant because the number of traits is set to 1 no matter what you have declared in the section.

EXAMPLE 4 (qxpak_4.par): A more complex analysis

This example comprises a two trait analysis (in posns. 4 and 5 of qxpak_4.dat file) with different models between traits. We are interested in testing whether qtl_1 is significant but, because there is still a qtl in the null model, the program does two qtl scans; qtl_1 is nested within sex, thus, the additive effect has two levels. qtl_2 is defined as mix_1a which means that there is a genetic variance associated but only for trait 1 because qtl_2 is not defined for trait 2. qtl_1 is not significant. Testing whether qtl_2 is significant is left as exercise.

EXAMPLE 5 (qxpak_5.par): Association study with molecular cov. matrix

APPENDIX 2: HISTORY

WHAT IS NEW IN QXPAK v. 5.03

- It allows for interaction within additive or dominant QTL effects.

WHAT IS NEW IN QXPAK v. 5.02

- Sex chromosome is specified in the parameter rather than in marker file
- Sex chromosome allowed for transposed files as well
- It allows for standardized or not molecular relationship matrices.

WHAT IS NEW IN QXPAK v. 5.0

- The possibility of including user defined matrices and, especially, tools to compute marker-bases relationship matrices that correct for structure in association studies or can be used, inefficiently though, for genome selection. See ran_mol option below for QTL modeling.
- Some options are deprecated like the color option or computing linkage disequilibrium.
- Some bugs are corrected.
- Higher dimensional (more than one locus in the model) scans are optimized to avoid repetitions of combinations.

WHAT IS NEW IN QXPAK v. 4.0

- Option to load all markers in memory when carrying out association studies (QTL defined as snp_* or ld_*) . See option MEMORY_RAM in parameter file section,, rather than writing and reading markers from files. Files zsnp* are no longer written.
- Option to read the marker file as transpose, i.e., when snps are arranged by rows instead of individuals. See marker file format.

Both these options makes it Qxpak much more efficient for large scale association studies. In an example with 6000 SNPs and 5000 individuals CPU time was reduced from 12’30’’ to 3’30’’ with the new version.

WHAT IS NEW IN QXPAK v. 3.2

- Bug when testing fixed effects and some missing data corrected.
- New option allowing to specifying easily that all haplotypes known

WHAT IS NEW IN QXPAK v. 3.0

- The most relevant feature is that it deals with epistasis. As usual for Qxpak, emphasis is in modeling flexibility without losing too much in computing efficiency. Epistasis can be accommodated between SNPs, or between fixed or random defined QTL. Standard and custom defined epistatic coefficients can be defined. See section ‘Modeling epistasis’ below.

WHAT IS NEW IN QXPAK v. 2.16

- Prints linkage disequilibrium, see PRINT_LD section
- Effects can be tested separately for each trait, see TEST section

WHAT IS NEW IN QXPAK v. 2.12

- Polar imprinting allowed for
- New section HAPLOTYPE_BLOCK_SIZE allows updating customized number of markers in phase sampling (may be useful if many markers in chromosome)

WHAT IS NEW IN QXPAK v. 2.10

- New format in section HAPLOTYPEOUTFILE
- New section PRINT_RESIDUALS allows printing of residuals and expected genetic values

WHAT IS NEW IN QXPAK v. 2.7

- Dummy pedigree file created if not specified. This dummy file assumes all individuals are unrelated.
- Imprinting: Imprinting has been added to the options of modeling QTL.
- ld_fix & ld_ran QTL modeling options has been added. These options are useful for association studies.
- SUMMARY_FILE section: A new summary file can be written for multi trait options.
- Multiple breed additive analyses: Crosses between multiple breed origins are allowed for.
- COLLAPSE_ORIGINS section: This option allows to treat all individuals from the same breed as clones in a random QTL model. Not tested.
- REUSE_VARIANCES: This option may speed computing.
- No recoding for individual is needed any longer: individual codes can be character type.
- New format that allows including a large number of markers.
- ACCURACY_POS section: the user can define that reliabilities are different for each trait and individual, or can analyze means of traits based on different number of records.
